# Exposing the structure of an Arctic food web

**DOI:** 10.1002/ece3.1647

**Published:** 2015-08-24

**Authors:** Helena K Wirta, Eero J Vesterinen, Peter A Hambäck, Elisabeth Weingartner, Claus Rasmussen, Jeroen Reneerkens, Niels M Schmidt, Olivier Gilg, Tomas Roslin

**Affiliations:** 1 Department of Agricultural Sciences, University of HelsinkiLatokartanonkaari 5, FI-00014, Helsinki, Finland; 2 Department of Biology, University of TurkuVesilinnantie 5, FI-20014, Turku, Finland; 3 Department of Ecology, Environment and Plant Sciences, Stockholm UniversitySE-106 91, Stockholm, Sweden; 4 Department of Bioscience, Aarhus UniversityNy Munkegade 114, DK–8000, Aarhus, Denmark; 5 Conservation Ecology Group, Groningen Institute for Evolutionary Life Sciences, University of GroningenP.O. Box 11103, 9700 CC, Groningen, The Netherlands; 6 Arctic Research Centre, Department of Bioscience, Aarhus UniversityFrederiksborgvej 399, DK-4000, Roskilde, Denmark; 7 Laboratoire Biogéosciences, UMR CNRS 6282, Université de Bourgogne6 Boulevard Gabriel, 21000, Dijon, France; 8 Groupe de Recherche en Ecologie Arctique16 rue de Vernot, 21440, Francheville, France

**Keywords:** *Calidris*, DNA barcoding, generalism, Greenland, Hymenoptera, molecular diet analysis, *Pardosa*, *Plectrophenax*, specialism, *Xysticus*

## Abstract

How food webs are structured has major implications for their stability and dynamics. While poorly studied to date, arctic food webs are commonly assumed to be simple in structure, with few links per species. If this is the case, then different parts of the web may be weakly connected to each other, with populations and species united by only a low number of links. We provide the first highly resolved description of trophic link structure for a large part of a high-arctic food web. For this purpose, we apply a combination of recent techniques to describing the links between three predator guilds (insectivorous birds, spiders, and lepidopteran parasitoids) and their two dominant prey orders (Diptera and Lepidoptera). The resultant web shows a dense link structure and no compartmentalization or modularity across the three predator guilds. Thus, both individual predators and predator guilds tap heavily into the prey community of each other, offering versatile scope for indirect interactions across different parts of the web. The current description of a first but single arctic web may serve as a benchmark toward which to gauge future webs resolved by similar techniques. Targeting an unusual breadth of predator guilds, and relying on techniques with a high resolution, it suggests that species in this web are closely connected. Thus, our findings call for similar explorations of link structure across multiple guilds in both arctic and other webs. From an applied perspective, our description of an arctic web suggests new avenues for understanding how arctic food webs are built and function and of how they respond to current climate change. It suggests that to comprehend the community-level consequences of rapid arctic warming, we should turn from analyses of populations, population pairs, and isolated predator–prey interactions to considering the full set of interacting species.

## Introduction

How interaction networks are structured comes with major implications for both their stability and dynamics (e.g., Thebault and Fontaine 2010). Yet, while large-scale variation in species richness is well-documented (Gaston 2000; Willig et al. 2003; Jenkins et al. 2013), we know substantially less about how local networks of biotic interactions are structured around the globe (Paine 1966; Lewinsohn and Roslin 2008).

The distribution of biotic interactions has often been discussed in terms of specialism versus generalism, that is, as a description of how many other nodes each node in the web interacts with. In this context, it has been suggested that species in species-rich communities are generally embedded in a lower number of biotic interactions than are species in species-poor communities, resulting in a higher generalism in species-poor communities at high latitudes (MacArthur 1972; Schemske 2009). Other studies suggest that a low diversity of resource taxa results in increasing specialism at high latitudes (Schleuning et al. 2012). Finally, some recent findings from networks of antagonistic interactions suggest that there may be no emergent relationship between the degree of specialism at a community-level and local species richness (Lewinsohn and Roslin 2008; Morris et al. 2014).

Clashing with the notion that specialism as such may be unrelated to – or even inversely related to – species diversity is the widely held belief that at the network level, food webs at high latitudes might be simple constructs (e.g., Elton 1927; Pimm 1982; Morin 1999; Krebs et al. 2003; Post et al. 2009; Legagneux et al. 2012). The very first description of a food web describes an arctic system with not only few taxa, but also sparse links between these taxa (Summerhayes and Elton 1923). Yet, this depiction of the web was strongly focused on vertebrates, and a further resolution of arthropod taxa within the web showed many more connections per species – both direct and indirect (Hodkinson and Coulson 2004). Indeed, arthropods form not only the main part of species richness, but also the main part of animal biomass in many regions (Strong et al. 1984; Wilson 1992), and several recent studies have pointed to invertebrates as forming the main part of arctic diversity (e.g., Danks 1992; Coulson and Refseth 2004; Jónsdóttir 2005; Fernandez-Triana et al. 2011; Várkonyi and Roslin 2013; H. K. Wirta, unpubl. data).

While the diversity of species-level nodes in arctic webs is thus beginning to emerge, our knowledge of trophic connections among these nodes is still scant – as resolving the trophic links among multiple taxa has been difficult. The main predator guilds of the arctic liquefy their prey prior to ingestion (spiders; Foelix 1996) or their digestion destroys visually identifiable prey parts (birds; Holmes 1966). Thus, previous observations of diet rely on scant observations of feeding events or invasive flushings of birds' crop (Major 1990).

As a result of both methodological and logistic challenges, we lack comprehensive descriptions of trophic interactions among multiple terrestrial guilds of the arctic – as we do for most other ecosystems. Yet, the link structure of a food web is essential. If the species or the links between them are left unresolved or only partially resolved, then the resultant web will be misleading with respect to any descriptor of biodiversity, food chain length, connectivity, and regulation of energy flow. In particular, the distribution of links per species – or specialism sensu *lato* – will be flawed, as will be all representations of compartmentalization within the web (Martinez 1991, 1993). As a maximal simplification of their importance, a set of straight and unconnected arctic food chains (signaling high “specialism”) will suggest vertical interactions, both direct and indirect, but little scope for horizontal indirect interactions through shared predators or resource taxa, while a dense and well-linked structure (high “generalism”) will allow indirect interactions traveling both through lower and higher trophic levels (Holt 1977; Chaneton and Bonsall 2000; Morris et al. 2004). Thus, a satisfactory understanding of a community's dynamics should be built on an appreciation of its trophic interaction structure.

Here, we offer the first highly resolved description of link structure for a major part of a terrestrial arctic food web. We used molecular techniques to reconstruct the web of trophic interactions between three predator guilds (insectivorous birds, spiders, and lepidopteran parasitoids) and their two quantitatively dominant prey taxa (Diptera and Lepidoptera). More specifically, we asked (1) whether the structure of the target community is more akin to a set of isolated food chains or a well-connected web; (2) whether individual predator taxa form separate modules within the web, or whether predators and predator guilds tap into the prey community of each other; and (3) what scope there is for indirect interactions traveling through this web. Overall, we discovered a densely linked food web. While based on a single highly resolved web, we hope that our finding will serve as both a benchmark and catalyst for shifting the focus of current research on arctic change from single species to networks of biotic interactions.

## Materials and Methods

To derive a comprehensive description of a high-arctic food web, three major predator guilds and two of their most important prey orders were combined in the same food web. For this purpose, the trophic interactions between the most abundant arthropod-feeding birds (three of eight locally breeding species), spiders (five of ten local species), and their prey species in the orders Diptera and Lepidoptera were resolved by molecular analyses of gut and fecal content. By joining these newly resolved trophic interactions with previously resolved parts of the same food web describing Lepidoptera–parasitoid interactions (involving 22 of 33 lepidopteran parasitoids occurring in the region; Wirta et al. 2014) and spider–prey interactions (three spider species; Wirta et al. 2015), the food webs of the different predator guilds were combined and compared, and the properties of the overall food web described.

### Study area

Our target region was the intensively studied area of the high-arctic Zackenberg Valley (74°30′N/21°00′W) within the Northeast Greenland National Park (for description, see Bay 1998; Meltofte and Rasch 2008; Sigsgaard et al. 2008). The diverse arthropod fauna of over 360 species includes no ants or ground beetles, and thus, spiders form the dominant arthropod predators. Diptera is the most species-rich order in the area, with close to 170 species, Hymenoptera the second with 59, and Lepidoptera the third with 21 species (H. K. Wirta, unpublished data). Diptera is also the most abundant order of the region (Høye and Forchhammer 2008), and Lepidoptera the locally dominant group of arthropod herbivores (Roslin et al. 2013).

### Predator species and sampling

**Figure 1:**
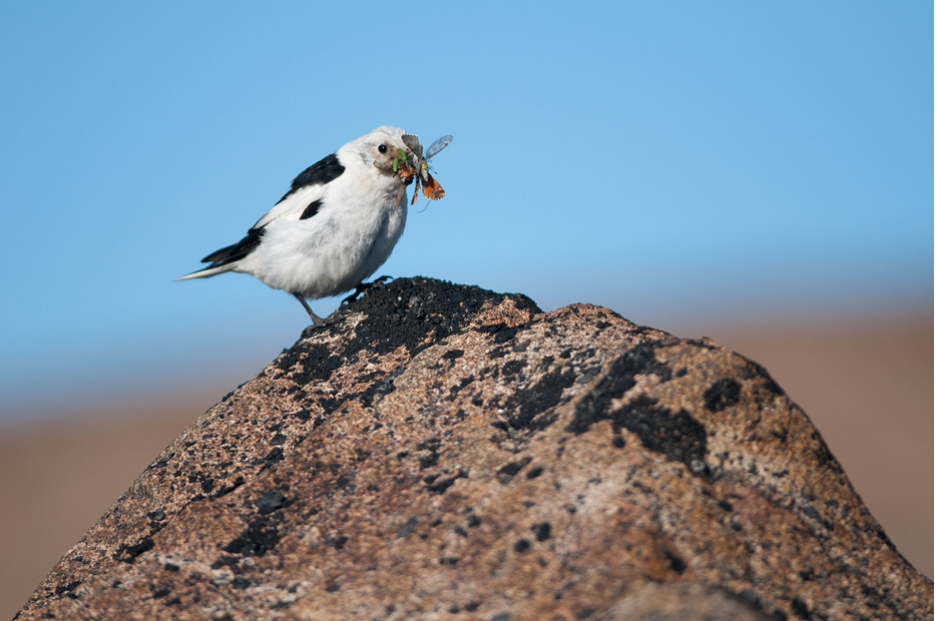
Concrete trophic interactions in the high arctic. Shown is a snow bunting (*Plectrophenax nivalis*) with its beak full of prey – with all recognizable items belonging to the two prey orders targeted here: Diptera and Lepidoptera. (Photograph by Juha Syväranta).

To describe trophic links involving the most important insectivores of the area, the diet of abundant arthropod-feeding birds of the region was examined: dunlin (*Calidris alpina* (Linnaeus)), sanderling (*C. alba* (Pallas)), and snow bunting (*Plectrophenax nivalis* (Linnaeus); Fig. 1; Hansen et al. 2010). Dunlin and sanderling are the two locally most common shorebirds; the snow bunting is common, too, and the single passerine species breeding regularly in the study area. Arthropods form the most important part of these birds' diet in summer (Cramp and Simmons 1983; Piersma et al. 1996). Trophic links involving these focal bird species were established by identifying prey DNA from fecal droppings (for details, see Appendix S1). To obtain adequate number of samples for analyses, samples of *C. alpina* and *C. alba* were also collected at the nearby locality of Hochstetter Forland (75^°^ 9′N/19^°^ 45′W). As preliminary analyses showed the bird diet of the two sites to be indistinguishable in terms of family-level prey contents (and the data too scarce to allow analyses at the species level), samples from the two sites were pooled for further analyses. The number of samples per species was constrained by sample availability, resulting in 14 droppings analyzed for *C. alpina*, 43 for *C. alba,* and 46 for *P. nivalis* (including fecal samples for both adults and chicks, with sample size too small for age-specific analyses).

To include spiders in the food web, five abundant species, representing all four families encountered in the study area: *Pardosa glacialis* (Thorell) (Lycosidae), *Xysticus deichmanni* Sorensen and *X. labradorensis* Keyserling (both in the family Thomisidae), *Emblyna borealis* (O. Pickard-Cambridge) (Dictynidae), and *Erigone arctica* White (Linyphiidae), were studied. The specimens were caught by live-catching pitfall traps and (mostly) by visual search and manual collecting. Trophic links were established by identifying prey remains from the gut of the spiders. For *E. arctica,* only ten individuals were analyzed, while for all other species, 120 individuals were examined. The two *Xysticus* species are considered here as a single compound taxon, as they cannot be reliably distinguished by external characters (Appendix S1; Wirta et al. 2015).

For lepidopteran parasitoids, the material studied by Wirta et al. (2014) was relied on. The specimens were caught by live-catching pitfall traps, hand-netting, and visual search. The species included comprise all abundant lepidopteran parasitoids of the region, as well as the vast majority (22 of 33) of the total lepidopteran parasitoid species pool (five of seven Hymenoptera: Braconidae; 14 of 21 Ichneumonidae; none of one Eulophidae; and three of three Diptera: Tachinidae species; Várkonyi and Roslin 2013; Wirta et al. 2014; G. Várkonyi pers. comm. 2014).

### Molecular analyses of the prey consumed

To identify the prey of birds, the DNA barcode region of mitochondrial CO1 (Hebert et al. 2003) was used. DNA was extracted from individual bird droppings, and each DNA extract was amplified twice with general arthropod primers. The PCR products were cleaned, tagged, and combined, then sequenced with a 318 chip on Ion Torrent Personal Genome Machine. Adapters and low-quality parts were trimmed from sequences, short reads removed, and the remaining sequences used to define operational taxonomic units (OTUs) as the prey (details of laboratory methods and processing of sequences in Appendix S1).

For spiders, three methods were used to examine the prey of *P. glacialis* and *Xysticus* spp., while *E. borealis* and *E. arctica* were examined with only the third method. For Methods 1 and 2, specimens of *P. glacialis* and *Xysticus* spp. were halved and one half was used for each method. In Method 1 (implemented by Wirta et al. 2015), DNA was extracted from individual spider halves, amplified with primers specific to Diptera and Lepidoptera, and sequenced directly by Sanger sequencing. In Method 2, halves for 9–15 individuals were pooled before DNA was extracted twice. DNA extracts were amplified with tagged Diptera–Lepidoptera-specific primers, and the PCR products were cleaned and sequenced with GS Junior (Roche 454; details in Appendix S1). For Method 3, as applied to all four studied spiders, the method designed and tested by Piñol et al. (2014) was adopted. DNA was extracted from halves of individuals singly, the DNA extracts were pooled into groups of 3–5 individuals, and the combined DNA extracts were amplified three times with general arthropod primers. The PCR products were cleaned, tagged, and run on a 318 chip on Ion Torrent Personal Genome Machine (details in Appendix S1). Support for consistency across methods is given in the supplementary information (Appendix S1).

For lepidopteran parasitoids, all trophic links detected across three methods implemented by Wirta et al. (2014; two molecular approaches and traditional rearing of parasitoids from host larvae) were adopted.

### Analyses of trophic interactions

As the methods used to reconstruct trophic links vary in terms of sampling unit and quantitative resolution, qualitative descriptors of food web structure are used (Banasek-Richter et al. 2009). The rationale is that a link established by any method offers proof of a feeding association between two taxa, whereas the type and reliability of information of the frequency of such interactions may differ between the methods employed to resolve them (cf. above).

To examine the accumulation of prey species used by each predator guild (and the extent to which each new predator species comes with a new set of prey species complementary to those already detected), rarefaction curves were constructed for prey species, treating individual predator species as samples within each predator guild. The program EstimateS, version 9.1.0 (Colwell 2013), was used for rarefactions, adopting the Bernoulli product model (Colwell et al. 2012) and 100 sample-order randomizations. To calculate how many prey species the total species pool of each predator guild would consume, the expected number of prey species used by the full set of predator species in each guild, as detected in the area (eight birds, ten spiders, and 33 lepidopteran parasitoids), was estimated. For this purpose, the rarefaction curves were extrapolated to the total number of species, using nonparametric methods (Colwell et al. 2012). This approach relies on the explicit assumption that the unsampled species are characterized by a similar prey range as the species included. This appears a reasonable approximation, as within the predator guilds where multiple species were studied (parasitoids: Wirta et al. 2014; spiders: Wirta et al. 2015), we see no indication of an association between diet breadth and predator abundance (beyond the one caused by sampling alone, with more prey species detected the more predator individuals were examined).

As a simple descriptor of prey use by different predators, we calculated the average number of dipteran and lepidopteran species directly connected to each species of spider, bird, and parasitoid (±SE). To visualize the architecture of the food web, qualitative webs were built by package bipartite (Dormann et al. 2009) implemented in program R (R Core Team 2012). The same package was used to depict the links between prey through shared predators by generalized overlap diagrams, as used by Roslin et al. (2013).

To examine whether different predator guilds – or some other species groups within the web – form compartments (i.e., sets of connected nodes unconnected to other nodes within the web), the number of compartments was calculated with package bipartite (Dormann et al. 2009). It was also examined whether the predator guilds – or some other species groups within the web – form modules (sets of highly connected nodes that are loosely connected to other such sets within the web by trophic interactions; Newman and Girvan 2004; Olesen et al. 2007). Modularity of the overall food web was estimated with the program MODULAR (Marquitti et al. 2014). For a given partitioning of a food web into modules, the modularity value *Q* is given by the difference between the observed fraction of edges connecting nodes in the same module and the expected fraction of edges connecting nodes in the same module if connections were to occur at random. The maximal *Q* value is given by the partition that best describes the present modules (Marquitti et al. 2014). To depict the web, the modularity value *Q*_B_ was chosen as best suited for bipartite data (Barber 2007) and optimized with default values (Marquitti et al. 2014). To test whether the proposed modules were stronger than those expected by chance, the proposed modularity was compared against two null models: the Erdós–Rényi model (Erdós and Rényi 1959) and “Null Model 2” of Bascompte et al. (2003), with 100 replicates for each.

In all of the analyses implemented, we depict the food web as two-layered or bipartite. This is a simplification of true trophic structure, as some of the predator species involved may occur at more than two strict layers, and because the prey taxa vary between first, second, and third-level consumers (e.g., Digel et al. 2014). Thus, the bipartite representation was chosen for convenience – and because our methods lack the resolution needed to fully resolve either interguild or intraguild predation among predators (e.g., a bird feeding on a spider feeding on a fly, or a spider feeding on another spider species, respectively; see Results for indications that both cases occur and Appendix S1 for relevant methodological restrictions). What we stress is that our key inferences regarding linkages, specialization, or compartmentalization of our target food web are robust to slight variation in the trophic level of individual taxa.

## Results

### The richness of prey species and trophic links

**Figure 2:**
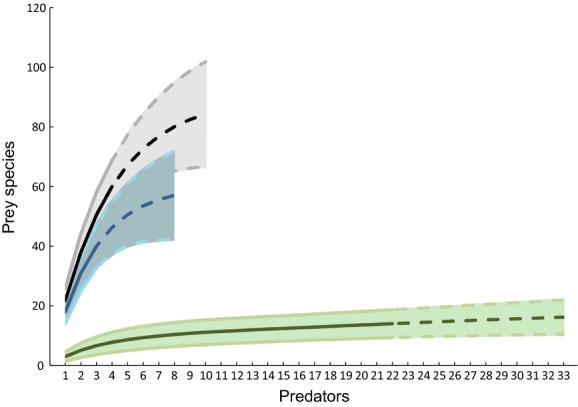
Numbers of prey taxa used by different predator guilds. Solid lines show accumulation curves based on empirical data (see Materials and Methods for details), and dashed lines extrapolations to the full number of predator species encountered in the area (eight birds, ten spiders, and 33 lepidopteran parasitoids). The lines in lighter color bordering the shaded area show the 95% confidence limits for each estimate. Blue lines represent birds, black ones spiders, and green ones lepidopteran parasitoids.

Overall, the reconstruction of food web structure revealed a total of 207 trophic links: 87 between spiders and their dipteran and lepidopteran prey, 54 between birds and their dipteran and lepidopteran prey, and 66 between lepidopteran parasitoids and their hosts (Fig.2, Table S1).

**Figure 3:**
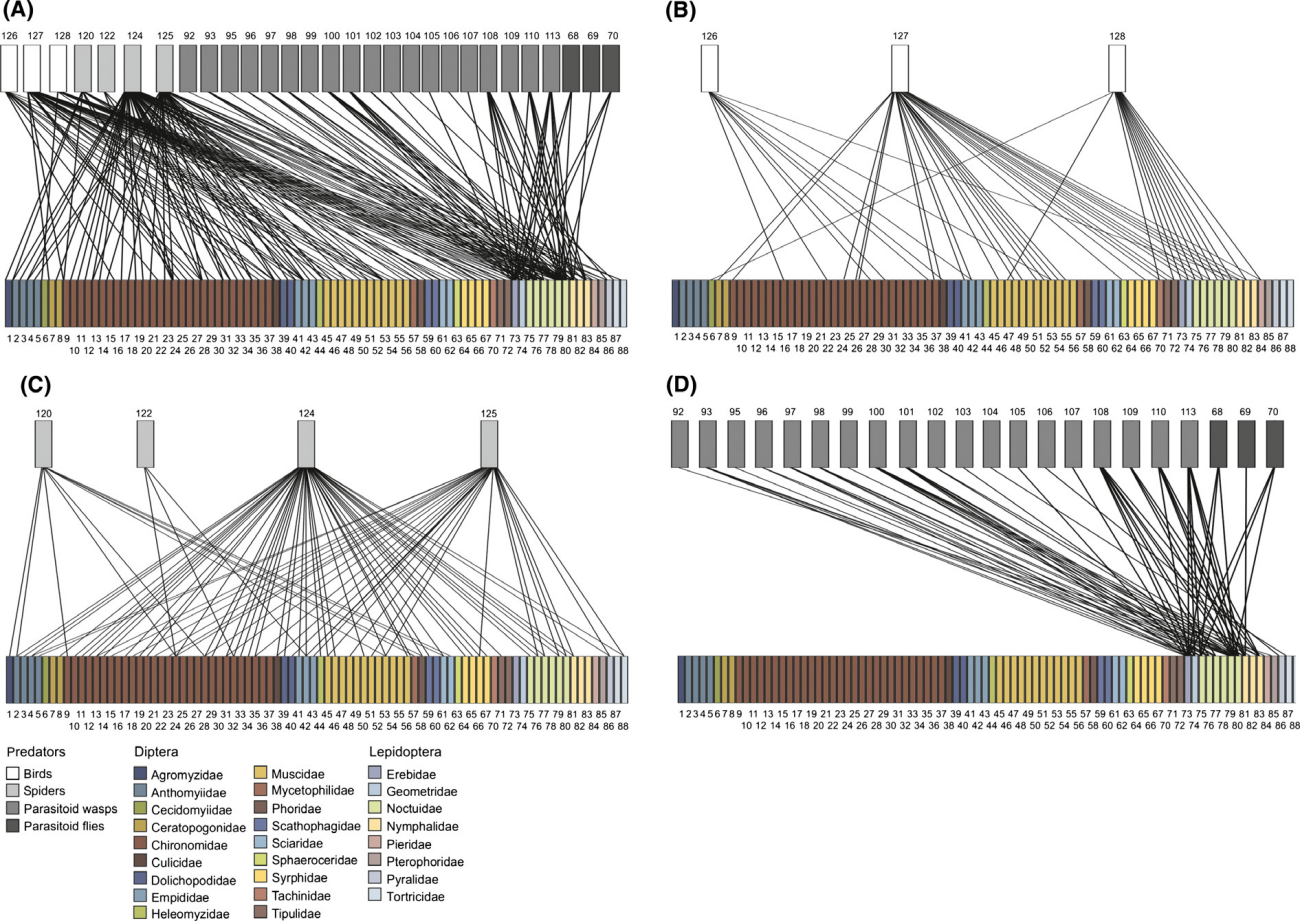
Qualitative food webs of the studied predators and their dipteran and lepidopteran prey, combining data from all methods used in the current study. The blocks in the upper row represent predator species and the blocks in the lower row the prey species. A line connecting a predator with a prey represents a detected predation event*. Here, different webs represent breakdown by predator guilds: (A) all predators combined, (B) birds, (C) spiders, and (D) lepidopteran parasitoids. The species are numbered as in Table 1, and different prey families are distinguished by different colors. *Note that the graph is qualitative and hence includes no information on the frequency of taxa or the interactions between them.

In terms of the taxonomic composition of prey use, 29 dipteran and 11 lepidopteran species in the droppings of the three focal bird species were detected. The spider species were found to consume 58 dipteran and eight lepidopteran species (current study and Wirta et al. 2015), whereas the parasitoids of Lepidoptera were detected to use fourteen host species (Wirta et al. 2014; Figs.2 and 3, Tables1 and S1).

**Table 1:** A full list of species encountered in the current study, with systematic affinity. The numbers offered in the right-hand column correspond to those used to identify species in the figures. Families and species are listed alphabetically, while classes and orders have been sorted to correspond with the figures. Listed first are therefore Diptera and Lepidoptera, as followed by other prey orders, and with predator orders last within classes. Some species were identified as by a species recorded in the BOLD (Ratnasingham and Hebert 2007), identified with a unique Barcode Index Number (Ratnasingham and Hebert 2013; shown, e.g., as BOLD:ACC1613)

Class	Order	Family	Species	No.
Insecta	Diptera	Agromyzidae	*Phytoliriomyza* sp.BOLD:ACC1613	1
		Anthomyiidae	*Egle groenlandica*	2
			*Fucellia pictipennis*	3
			*Paradelia arctica*	4
			*Zaphne frontata*	5
		Cecidomyiidae	*Cecidomyiidae* sp. BOLD:AAN5271	6
		Ceratopogonidae	*Ceratopogonidae* sp. BOLD:AAO7733	7
			*Culicoides* sp. BOLD:AAM6201	8
		Chironomidae	*Allocladius nanseni*	9
			*Chironomidae* sp.BOLD:ACC5452	10
			*Chironomidae* sp. unknown 1	11
			*Chironomus hyperboreus*	12
			*Cladotanytarsus mancus*	13
			*Cladotanytarsus pallidus*	14
			*Halocladius variabilis*	15
			*Hydrobaenus fusistylus*	16
			*Limnophyes* cf. *brachytomus*	17
			*Limnophyes minimus*	18
			*Limnophyes* sp. A	19
			*Metriocnemus* sp. 1ES	20
			*Microtendipes pedellus*	21
			*Orthocladius decoratus*	22
			*Orthocladius frigidus*	23
			*Paraphaenocladius impensus*	24
			*Procladius* cf. *crassinervis*	25
			*Procladius crassinervis*	26
			*Psectrocladius barbimanus*	27
			*Smittia edwardsi*	28
			*Smittia extrema*	29
			*Smittia* sp. 16ES	30
			*Smittia* sp. 25ES	31
			*Smittia* sp. 2ES	32
			*Smittia* sp. 6ES	33
			*Smittia* sp. BOLD:ABA7010	34
			*Smittia* sp. BOLD:ABA7011	35
			*Tanytarsus* sp. BOLD:ACB5329	36
			*Tanytarsus* sp. BOLD:ACB5827	37
		Culicidae	*Aedes impiger/nigripes*	38
		Dolichopodidae	*Dolichopus longitarsis*	39
			*Dolichopus ungulatus*	40
		Empididae	*Rhamphomyia filicauda*	41
			*Rhamphomyia nigrita*	42
		Ephydridae	*Lamproscatella sibilans*	43
		Heleomyzidae	*Neoleria prominens*	44
		Muscidae	*Drymeia groenlandica*	45
			*Drymeia segnis*	46
			*Musca domestica*	47
			*Phaonia bidentata*	48
			*Spilogona almqvistii*	49
			*Spilogona dorsata*	50
			*Spilogona megastoma*	51
			*Spilogona melanosoma*	52
			*Spilogona sanctipauli*	53
			*Spilogona tornensis*	54
			*Spilogona tundrae*	55
			*Spilogona zaitzevi*	56
		Mycetophilidae	*Exechia frigida*	57
		Phoridae	*Megaselia cirriventris*	58
		Scathophagidae	*Gonarcticus arcticus*	59
			*Scathophaga nigripalpis*	60
		Sciaridae	*Lycoriella riparia*	61
			*Scatopsciara atomaria*	62
		Sphaeroceridae	*Spelobia* sp. BOLD:AAN6408	63
		Syrphidae	*Baccha elongata*	64
			*Eupeodes punctifer*	65
			*Parasyrphus tarsatus*	66
			*Platycheirus groenlandicus*	67
		Tachinidae	*Exorista thula*	68
			*Periscepsia stylata*	69
			*Peleteria aenea*	70
		Tipulidae	*Nephrotoma lundbecki*	71
			*Tipula arctica*	72
Insecta	Lepidoptera	Erebidae	*Gynaephora groenlandica*	73
		Geometridae	*Entephria polata*	74
		Noctuidae	*Apamea zeta*	75
			*Euxoa adumbrata drewseni*	76
			*Polia richardsoni*	77
			*Rhyacia quadrangula*	78
			*Sympistis nigrita zetterstedtii*	79
			*Syngrapha parilis*	80
		Nymphalidae	*Boloria chariclea*	81
			*Boloria polaris*	82
			*Boloria chariclea* or *polaris*	83
		Pieridae	*Colias hecla*	84
		Pterophoridae	*Stenoptilia mengeli*	85
		Pyralidae	*Pyla fusca*	86
		Tortricidae	*Olethreutes inquietana*	87
			*Olethreutes mengelana*	88
	Coleoptera	Dermestidae	*Anthrenus verbasci*	89
	Hemiptera	Aphididae	*Myzus persicae*	90
		Lygaeidae	*Nysius groenlandicus*	91
	Hymenoptera	Braconidae	*Dolichogenidea* cf. *sicaria*	92
			*Microplitis lugubris*	93
			*Praon brevistigma*	94
			*Protapanteles fulvipes*	95
		Ichneumonidae	*Aoplus groenlandicus*	96
			*Buathra laborator*	97
			*Campoletis horstmanni*	98
			*Campoletis rostrata*	99
			*Cotesia* spp.	100
			*Cryptus arcticus*	101
			*Cryptus leechi*	102
			*Diadegma majale*	103
			*Exochus pullatus*	104
			*Gelis maesticolor*	105
			*Hormius moniliatus*	106
			*Hyposoter deichmanni*	107
			*Hyposoter frigidus*	108
			*Ichneumon discoensis*	109
			*Mesochorus* n. sp.	110
			*Neurateles* sp. 1 ZERO	111
			*Phygadeuon solidus*	112
			*Pimpla sodalis*	113
		Tenthredinidae	*Amauronematus nitidipleuris*	114
Collembola	Entomobryomorpha	Entomobryidae	*Entomobryidae* sp. BOLD:AAI5219	115
Clitellata	Haplotaxida	Enchytraeidae	*Bryodrilus diverticulatus*	116
Arachnida	Sarcoptiformes	Ceratozetidae	*Diapterobates* sp. nov.BOLD:ACH0107	117
	Trombidiformes	Lebertiidae	*Lebertiidae* sp. BOLD:ACK2963	118
		Penthaleidae	*Penthaleidae* sp. BOLD:AAN6605	119
	Araneae	Dictynidae	*Emblyna borealis*	120
		Linyphiidae	*Collinsia spetsbergensis*	121
			*Erigone arctica*	122
			*Hilaira vexatrix*	123
		Lycosidae	*Pardosa glacialis*	124
		Thomisidae	*Xysticus deichmanni* & *labradorensis*	125
Aves	Charadriiformes	Scolopacidae	*Calidris alpina*	126
			*Calidris alba*	127
	Passeriformes	Emberizidae	*Plectrophenax nivalis*	128

Naturally, the number of links observed will offer a subset of the total web of the area, as a subset of species within each predator guild was sampled and two prey orders were focused on. Extrapolating from the predator species examined to the full pool of predators of the studied groups present in the region suggests that eight locally breeding bird species will use a total of ca 57 species of dipteran and lepidopteran prey (95% confidence limits of 42.2–71.8), that ten spider species will use a full 84 species of Diptera and Lepidoptera (66.7–101.9), and that 33 parasitoid species will use a total of 16 species of Lepidoptera (10.4–21.9).

### The structure of the overall web structure

Altogether, the large set of links observed results in a highly linked structure, with each spider taxon directly connected to an average of 18 ± 7.8 dipteran and 4 ± 2.2 lepidopteran species, each bird to 11 ± 5.5 dipteran and 7 ± 6.7 lepidopteran species, and each parasitoid to 3 ± 0.5 lepidopteran species. When combined across taxa, we find a web that is highly connected through host and prey as well as predator and parasitoid species (Figs.2 and 3).

We found no evidence of real compartmentalization in the overall web, with one big compartment including all but four species. The only trace of substructuring related to two separate compartments consisting of one lepidopteran parasitoid and one lepidopteran species each. These two species pairs represent poorly sampled parts of the web, as only one larva of both these lepidopteran species was analyzed for parasitoid contents and the parasitoid species detected within these larvae had never been sampled for gut contents (Wirta et al. 2014). Thus, the discreteness of these compartments could be generated by undersampling alone, and overall, the different predator guilds and their prey were well-connected to each other forming a single compartment. The lack of compartmentalization was further supported by the analysis of modularity, with *Q*_B_ being 0.49 and far from significant as based on comparison with the two null models (*P* = 0.77 for the Erdós–Rényi model and *P* = 0.23 for “Null Model 2”).

The generalized overlap diagrams reveal an extreme incidence of shared predators among individual dipteran and lepidopteran prey species (Fig.3). Yet, the different predator guilds contribute differently to this pattern. Spiders have the highest number of shared prey taxa, while birds offer more links among Lepidoptera than Diptera. Lepidopteran parasitoids tie dense links among the lepidopteran part of the prey community. While part of these differences may arise from the differences in methods used for the different guilds, the different predator taxa do offer different scope for mediating indirect interactions between parts of the prey community, but each part of the web is still connected to every other part of the web through at least some indirect links.

Adding to the diversity of trophic links, the predator guilds were also found to feed on each other. While we here focus on dipteran and lepidopteran prey, part of the methods (cf. Text S1) offered evidence for birds consuming spiders and lepidopteran parasitoids, for spiders feeding on other spiders (Appendix S1, Fig. S3), and for birds and spiders feeding on other predatory taxa (see the complete matrix of trophic interactions detailed in Table S1).

## Discussion

This study shows the members of an arctic food web to be linked to each other through versatile trophic interactions. Using a range of complementary methods, we demonstrate that our target food web includes a large amount of feeding relations among predators and prey and that this linking offers ample scope for indirect interactions traveling through the web. Overall, this benchmark dissection of a high-arctic food web paves the way for a new view on arctic communities – with implications for how we should be monitoring arctic communities under progressing climate change. Below, we will address each of these findings in turn.

### A densely linked arctic food web

Many descriptions of arctic food webs have been focused on a relatively few vertebrate taxa (e.g., Summerhayes and Elton 1923; Krebs et al. 2003; Legagneux et al. 2012), but recent studies have exposed the diversity of arthropod nodes within these webs (Coulson and Refseth 2004; Jónsdóttir 2005; Fernandez-Triana et al. 2011; Várkonyi and Roslin 2013). By resolving the links between nodes, we identify the arthropods as forming the majority of connections. Thus, neglecting or failing to resolve these taxa would result in a misrepresentation of all aspects of network structure (Martinez 1991, 1993).

**Figure 4:**
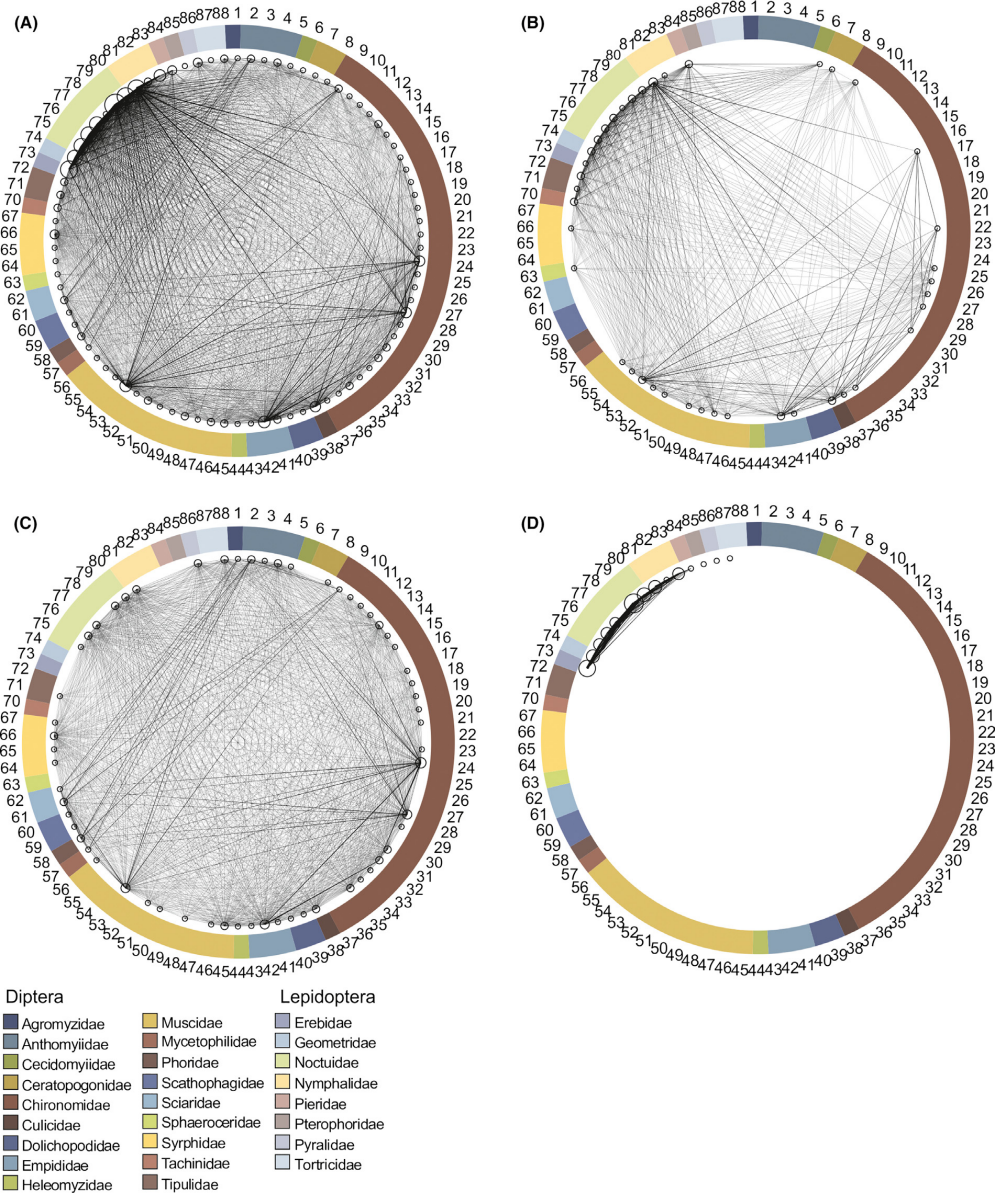
Qualitative generalized overlap diagrams showing shared predators among dipteran and lepidopteran prey showing the prey species with potential for indirect interactions. In each panel, the small circles on the perimeter represent prey species (numbered as in Table 1), and families are identified by colors on the surrounding circle. Each line connecting two prey species (small circles) represents a predator species shared among the respective prey species, thus revealing the potential for indirect interactions among the species linked together. The size of the circle is proportional to how many times this prey species was detected among the predators, with the strength of the line proportional to how many times a predator species was found to use the two prey species. Different panels represent different predator guilds: (A) all predators combined, (B) birds, (C) spiders, and (D) lepidopteran parasitoids.

Clearly, the dominant arthropod feeders of the Zackenberg food web, that is, birds and spiders, are generalist hunters with a potentially broad diet wherever they occur. Thus, our primary claim is not that their diet would be wider in the arctic than elsewhere (but see Wirta et al. 2015) – but that feeding interactions involving these generalist taxa contribute strongly to the overall arctic interaction web, thus dictating its emergent structure (cf. Figs.4, Table1). Nonetheless, generalism may indeed be a trait favored in the arctic, where low productivity and large variation in resource availability through time can both be limiting the potential for specialism (Høye and Forchhammer 2008). Thus, both the overall composition of the predator community and selection on life-history traits may contribute to the overall structure of the web – in so far unknown proportions.

Adding to dietary plasticity within the arctic community is the scope for cannibalism among the spiders as a key predator guild. While our current methods fall short of accurately resolving either inter- or intraguild predation among predators (Appendix S1), we know from both field observations (Visakorpi et al., 2015) and stray records obtained in the current study (Appendix S1, Fig. S3) that birds will at least sometimes consume spiders and lepidopteran parasitoids, spiders will eat other spider species, and both birds and spiders will feed on other predatory taxa (see the complete matrix of trophic interactions detailed in Table S1). Intraspecific predation is common in spiders and has often been observed within the species studied here (Visakorpi et al., 2015; Appendix S1, Fig. S3). Such patterns may affect community dynamics: Within species, the consumption of conspecifics may form an additional food supply, but from the perspective of other species, it also comes with the positive side effect of erasing competitors (for predators) or predators (for prey: Wise 2006). Overall, a generalist diet might then be vital for reducing both competition and risks of starvation in the harsh arctic environments (Riechert and Lockley 1984; Toft and Wise 1999).

### How are arctic interaction webs structured?

Our exploration of the Zackenberg food web offers no support for separate food chains or for separate compartments or modules within the web – but evidence for high connectivity across predators and prey species. In a web like the one observed, the links connecting all species offer versatile scope for indirect interactions. Modular patterns in food webs increase the stability of the overall network, retaining the impacts of a perturbation within a single module and minimizing impacts on other modules (Krause et al. 2003; Teng and McCann 2004). In a similar vein, compartmentalization limits the changes to have an effect only within the compartment and thus increases persistence to disturbances for the other compartments (Stouffer and Bascompte 2011).

Interestingly, different features have been proposed to promote stability in networks of different interactions (Thebault and Fontaine 2010): While in trophic networks (i.e., food webs), stability is enhanced by compartmented and weakly connected architectures (see above) and stability in mutualistic networks is enhanced by the opposite feature: a highly connected architecture. Yet, both types of networks seem well-connected in our high-arctic setting (cf. Appendix S2). Before rushing to infer the dynamical properties of the target network from these descriptions, we should stress just how strongly the perceived structure of a network will depend on the methods used to reconstruct it (Wirta et al. 2014). As the current description is based on methods shown to offer unusual resolution (Wirta et al. 2014), we should regard it as a new benchmark for future comparisons, not as a data point to conveniently slip into previous patterns.

In the current, well-resolved web, we can expect a change in one species to affect a multitude of others through direct and indirect interactions traveling through the web. As an example, while the parasitoids of Lepidoptera are confined to feeding on lepidopteran host taxa, the current study shows this part of the web (as described by Wirta et al. 2014) to be well-connected to all other parts of the web. Thus, a parasitoid species suppressing the population of a butterfly species will indirectly affect a spider population feeding on the same butterfly.

Our findings from the current combination of subwebs of three predator guilds come with general implications for any study aiming to reconstruct interaction structure in nature. The subwebs centered on individual predator guilds form no clear substructures within the overall web. Had we not combined the predator guilds, we would have missed a major part of indirect interactions possibly affecting each prey species – as these interactions are by no means confined to a single guild (see also Appendix S2 and discussion therein). Yet, only rarely do studies of food webs explore multiple predator guilds at the same time (but see Pocock, Evans & Memmott 2012). Quite the contrary, most studies of terrestrial systems conducted to date have focused on single guilds of predators (e. g. Albrecht et al. 2007; Tylianakis et al. 2007; van Veen et al. 2008; Henson et al. 2009). Thus, if guilds within the same food web are strongly linked, then the examination of a small proportion of a large food web may lead to erroneous conclusions regarding the relative role of interactions within the focal part of the web. The molecular methods now adopted facilitate comparisons among different types of taxa and interactions (Clare 2014; Hrcek and Godfray 2015; Symondson and Harwood 2014). Importantly though, the current study comes with no quantification of the relative strength of individual trophic links. This should be the logical next step to target with improved techniques.

### A match with large-scale patterns

The current study offers an in-depth description of a single type of biotic interactions (feeding associations) for a single locality in Northeast Greenland. Elsewhere, we have demonstrated that our impression of network structure may change with the methods employed to reconstruct the web and that molecular methods offer highly sensitive tools for resolving interaction structure (Wirta et al. 2014). Other studies have, on their part, shown that different types of interaction webs may be structured by different influences (e.g., Fontaine et al. 2009; Guimarães et al. 2011; Schleuning et al. 2012; Legagneux et al. 2014; Morris et al. 2014), precluding direct comparisons among interactions of different types. Thus, we are clearly in no position to directly compare the current food web with other food webs reconstructed elsewhere. Until molecular-based representations of web structure become available for other parts of the arctic (and for other parts of the world), the current network should be seen as a single data point – but as such, it forms an important benchmark for future descriptions of food webs.

What adds credence to preliminary inference that food webs from high latitudes are overall no less connected than ones from low latitudes is a recent meta-analysis comparing host–parasitoid food webs from different latitudes but generated by a single technique (the rearing of parasitoids from hosts; Morris et al. 2014). While such a technique will fail to resolve a significant proportion of all trophic links in the web (Wirta et al. 2014), they offer mutually commensurate depictions of food web structure. Here, Zackenberg was included, and the resultant pattern across latitudes supports no general trends in linkage structure toward the poles (Morris et al. 2014).

The lack of increase in specialism toward the poles matches the patterns reported for other interaction types, which we also show to form well-linked webs in our study region (Appendix 2). Comparisons between tropical and temperate latitudes offer no evidence of latitudinal differences in the specialism of antagonistic interactions of herbivores and plants (Beaver 1979; Fiedler 1998; Novotny et al. 2002, 2006; Lewinsohn and Roslin 2008), or suggest an increase in specialism toward the equator (Dyer et al. 2007). An increase in specialism toward the equator has been also found in mutualistic interactions of plants and pollinators (Olesen and Jordano 2002; Armbruster 2006; Dalsgaard et al. 2011; Trojelsgaard and Olesen 2013), but such latitudinal trends sometimes disappear once sampling bias (Ollerton and Cranmer 2002; Vázquez and Stevens 2004) or differences in plant diversity (Ollerton et al. 2006) have been accounted for. Methods, taxonomic coverage, and level of resolution vary widely among recent studies, with the resultant data points being heavily biased toward low and intermediated latitudes (Fontaine et al. 2009; Schleuning et al. 2012; Morris et al. 2014). Yet, with Zackenberg as the only data point north of the arctic circle, they seem to jointly attest against any general simplification of biotic interaction structure toward the poles (but see Schleuning et al. 2012).

### Implications

To date, our exploration of the local food web of Zackenberg has found support for a dense link structure and low specialism in antagonistic interactions including multiple insectivorous predators (current study; Wirta et al. 2014, 2015) as well as herbivores and plants (Appendix S2; Roslin et al. 2013), but also in mutualistic interactions among pollinators and plants (Appendix S2; Rasmussen et al. 2013). In consequence, these two traits appear to be features not of any particular interaction type, but of many types of interactions in the community studied. A full appreciation of arctic food web complexity will require the simultaneous assessment of multiple interaction types. Thus, what our study ultimately suggests is that to understand the community-level consequences of rapid arctic warming, we should turn from analyses of populations, population pairs, and isolated predator–prey couplings to considering all the species interacting within arctic communities.

## Data Accessibility

The sequences obtained for the current study are stored in Dryad (doi:10.5061/dryad.cv8cr).

## Supporting Information

**Table S1.** Matrix of trophic interactions.

**Appendix S1.** Detailed methods and additional results.

**Appendix S2.** Further components of the Zackenberg interaction web.

## References

[b1] Albrecht M, Duelli P, Schmid B, Muller CB (2007). Interaction diversity within quantified insect food webs in restored and adjacent intensively managed meadows. J. Anim. Ecol.

[b2] Armbruster WS, Waser NM, Ollerton J (2006). Evolutionary and ecological aspects of specialized pollination: views from the arctics to the tropics. Plant-pollinator interactions: from specialization to generalization.

[b3] Banasek-Richter C, Bersier LF, Cattin MF, Baltensperger R, Gabriel JP, Merz Y (2009). Complexity in quantitative food webs. Ecology.

[b4] Barber MJ (2007). Modularity and community detection in bipartite networks. Phys. Rev. E.

[b5] Bascompte J, Jordano P, Melian CJ, Olesen JM (2003). The nested assembly of plant-animal mutualistic networks. Proc. Natl Acad. Sci. USA.

[b6] Bay C (1998). Vegetation mapping of Zackenberg valley, Northeast Greenland.

[b7] Beaver RA (1979). Host specificity of temperate and tropical animals. Nature.

[b8] Chaneton EJ, Bonsall MB (2000). Enemy-mediated apparent competition: empirical patterns and the evidence. Oikos.

[b9] Clare EL (2014). Molecular detection of trophic interactions: emerging trends, distinct advantages, significant considerations and conservation applications. Evol. Appl.

[b10] Colwell RK (2013). http://purl.oclc.org/estimates.

[b11] Colwell RK, Chao A, Gotelli NJ, Lin SY, Mao CX, Chazdon RL (2012). Models and estimators linking individual-based and sample-based rarefaction, extrapolation and comparison of assemblages. J. Plant Ecol.

[b12] Coulson SJ, Strøm H, Goldman H, Refseth D (2004). The terrestrial and freshwater invertebrate fauna of Svalbard (and Jan Mayen): a species and reference checklist. A catalogue of the terrestrial and marine animals.

[b13] Cramp S, Simmons KEL (1983). Handbook of the birds of Europe, the Middle East and North Africa. Volume III Waders to gulls.

[b14] Dalsgaard B, Magård E, Fjeldså J, Martín González AM, Rahbek C, Olesen JM (2011). Specialization in plant-hummingbird networks is associated with species richness, contemporary precipitation and quaternary climate-change velocity. PLoS ONE.

[b15] Danks HV (1992). Arctic insects as indicators of environmental-change. Arctic.

[b16] Digel C, Curtsdotter A, Riede J, Klarner B, Brose U (2014). Unravelling the complex structure of forest soil food webs: higher omnivory and more trophic levels. Oikos.

[b17] Dormann CF, Fründ J, Blüthgen N, Gruber B (2009). Indices, graphs and null models: analyzing bipartite ecological networks. Open Ecol. J.

[b18] Dyer LA, Singer MS, Lill JT, Stireman JO, Gentry GL, Marquis RJ (2007). Host specificity of Lepidoptera in tropical and temperate forests. Nature.

[b19] Elton CS (1927). Animal ecology.

[b20] Erdós P, Rényi A (1959). On random graphs. Publicationes Mathematicae Debrecen.

[b21] Fernandez-Triana J, Smith MA, Boudreault C, Goulet H, Hebert PDN, Smith AC (2011). A poorly known high-latitude parasitoid wasp community: unexpected diversity and dramatic changes through time. PLoS ONE.

[b22] Fiedler K (1998). Diet breadth and host plant diversity of tropical- vs. temperate-zone herbivores: South-East Asian and West Palaearctic butterflies as a case study. Ecol. Entomol.

[b23] Foelix RF (1996). Biology of Spiders.

[b24] Fontaine C, Thebault E, Dajoz I (2009). Are insect pollinators more generalist than insect herbivores?. Proc. Biol. Sci.

[b25] Gaston KJ (2000). Global patterns in biodiversity. Nature.

[b26] Guimarães PRJ, Jordano P, Thompson JN (2011). Evolution and coevolution in mutualistic networks. Ecol. Lett.

[b27] Hansen J, Hansen LH, Boesgaards K, Albert K, Svendsen S, Hoffman Hansen S, Magelund Jensen L, Rasch R (2010). Zackenberg Basic: the BioBasis programme. Zackenberg Ecological Research Operations: 15th Annual Report 2009.

[b28] Hebert PDN, Ratnasingham S, deWaard JR (2003). Barcoding animal life: cytochrome *c* oxidase subunit 1 divergences among closely related species. Proc. R. Soc. B Biol. Sci.

[b29] Henson KSE, Craze PG, Memmott J (2009). The restoration of parasites, parasitoids, and pathogens to heathland communities. Ecology.

[b30] Hodkinson ID, Coulson SJ (2004). Are high Arctic terrestrial food chains really that simple? The Bear Island food web revisited. Oikos.

[b31] Holmes RT (1966). Feeding ecology of the red-backed sandpiper (*Calidris alpina*) in Arctic Alaska. Ecology.

[b32] Holt RD (1977). Predation, apparent competition, and the structure of prey communities. Theor. Popul. Biol.

[b33] Høye TT, Forchhammer MC (2008). Phenology of high-arctic arthropods: effects of climate on spatial, seasonal, and inter-annual variation. Adv. Ecol. Res.

[b34] Hrcek J, Godfray HCJ (2015). What do molecular methods bring to host-parasitoid food webs?. Trends Parasitol.

[b35] Jenkins CN, Pimm SL, Joppac LN (2013). Global patterns of terrestrial vertebrate diversity and conservation. Proc. Natl Acad. Sci. USA.

[b36] Jónsdóttir IS (2005). Terrestrial ecosystems on Svalbard: heterogeneity, complexity and fragility from an Arctic island perspective. Biol. Environ. Proc. Roy. Irish Acad.

[b37] Krause AE, Frank KA, Mason DM, Ulanowicz RE, Taylor WW (2003). Compartments revealed in food-web structure. Nature.

[b38] Krebs CJ, Danell K, Angerbjorn A, Agrell J, Berteaux D, Brathen KA (2003). Terrestrial trophic dynamics in the Canadian Arctic. Can. J. Zool.

[b39] Legagneux P, Gauthier G, Berteaux D, Bety J, Cadieux MC, Bilodeau F (2012). Disentangling trophic relationships in a High Arctic tundra ecosystem through food web modeling. Ecology.

[b40] Legagneux P, Gauthier G, Lecomte N, Schmidt NM, Reid D, Cadieux M-C (2014). Arctic ecosystem structure and functioning shaped by climate and herbivore body size. Nat. Clim. Chang.

[b41] Lewinsohn TM, Roslin T (2008). Four ways towards tropical herbivore megadiversity. Ecol. Lett.

[b42] MacArthur RH (1972). Geographical ecology: patterns in the distribution of species.

[b43] Major RE (1990). Stomach flushing of an insectivorous bird – an assessment of differential digestibility of prey and the risk to birds. Aust. Wildl. Res.

[b44] Marquitti FMD, Guimaraes PR, Pires MM, Bittencourt LF (2014). MODULAR: software for the autonomous computation of modularity in large network sets. Ecography.

[b45] Martinez ND (1991). Artifacts or attributes? Effects of resolution on the Little Rock Lake food web. Ecol. Monogr.

[b46] Martinez ND (1993). Effects of resolution on food web structure. Oikos.

[b47] Meltofte H, Rasch M (2008). The study area at Zackenberg: high Arctic ecosystem dynamics in a changing climate: ten years of monitoring and research at Zackenberg research station, Northeast Greenland. Adv. Ecol. Res.

[b48] Morin PJ (1999). Community ecology.

[b49] Morris RJ, Lewis OT, Godfray HC (2004). Experimental evidence for apparent competition in a tropical forest food web. Nature.

[b50] Morris RJ, Gripenberg S, Lewis OT, Roslin T (2014). Antagonistic interaction networks are structured independently of latitude and host guild. Ecol. Lett.

[b51] Newman MEJ, Girvan M (2004). Finding and evaluating community structure in networks. Phys. Rev. E.

[b52] Novotny V, Basset Y, Miller SE, Weiblen GD, Bremer B, Cizek L (2002). Low host specificity of herbivorous insects in a tropical forest. Nature.

[b53] Novotny V, Drozd P, Miller SE, Kulfan M, Janda M, Basset Y (2006). Why are there so many species of herbivorous insects in tropical rainforests?. Science.

[b54] Olesen JM, Jordano P (2002). Geographic patterns in plant-pollinator mutualistic networks. Ecology.

[b55] Olesen JM, Bascompte J, Dupont YL, Jordano P (2007). The modularity of pollination networks. Proc. Natl Acad. Sci. USA.

[b56] Ollerton J, Cranmer L (2002). Latitudinal trends in plant-pollinator interactions: are tropical plants more specialised?. Oikos.

[b57] Ollerton J, Johnson SD, Waser NM, Ollerton J, Hingston AB (2006). Geographical variation in diversity and specificity of pollination systems. Plant-pollinator interactions: from specialization to generalization.

[b58] Paine RT (1966). Food web complexity and species diversity. Am. Nat.

[b59] Piersma T, van Gils JA, Del Hoyo J, Elliott A, Sargatal J, Wiersma P (1996). Family Scolopacidae (sandpipers, snipes and phalaropes). Handbook of the birds of the world.

[b60] Pimm SL (1982). Food webs.

[b61] Piñol J, San Andrés V, Clare EL, Symondson WOC (2014). A pragmatic approach to the analysis of diets of generalist predators: the use of next-generation sequencing with no blocking primers. Mol. Ecol. Resour.

[b600] Pocock MJO, Evans DM, Memmott J (2012). The robustness and restoration of a network of ecological networks. Science.

[b62] Post E, Forchhammer MC, Bret-Harte MS, Callaghan TV, Christensen TR, Elberling B (2009). Ecological dynamics across the Arctic associated with recent climate change. Science.

[b63] R Core Team (2012). R: a language and environment for statistical computing.

[b64] Rasmussen C, Dupont YL, Mosbacher JB, Trøjelsgaard K, Olesen JM (2013). Strong Impact of Temporal Resolution on the Structure of an Ecological Network. PLoS ONE.

[b65] Ratnasingham S, Hebert PDN (2007). BOLD: the barcode of life data system. Mol. Ecol. Notes.

[b66] Ratnasingham S, Hebert PDN (2013). A DNA-based registry for all animal species: the Barcode Index Number (BIN) system. PLoS ONE.

[b67] Riechert SE, Lockley T (1984). Spiders as biological control agents. Annu. Rev. Entomol.

[b68] Roslin T, Wirta H, Hopkins T, Hardwick B, Várkonyi G (2013). Indirect interactions in the High Arctic. PLoS ONE.

[b69] Schemske DW, Butlin R, Bridle J, Schluter D (2009). Biotic interactions and speciation in the tropics. Speciation and patterns of diversity.

[b70] Schleuning M, Frund J, Klein AM, Abrahamczyk S, Alarcon R, Albrecht M (2012). Specialization of mutualistic interaction networks decreases toward tropical latitudes. Curr. Biol.

[b71] Sigsgaard C, Rasmussen L, Cappelen J, Hinkler J, Mernild SH, Petersen D (2008). Present-day climate at Zackenberg (High Arctic ecosystem dynamics in a changing climate: ten years of monitoring and research at Zackenberg research station, Northeast Greenland). Adv. Ecol. Res.

[b72] Stouffer DB, Bascompte J (2011). Compartmentalization increases food-web persistence. Proc. Natl Acad. Sci. USA.

[b73] Strong DR, Lawton JH, Southwood SR (1984). Insects on plants: community patterns and mechanisms.

[b74] Summerhayes VS, Elton CS (1923). Contributions to the ecology of Spitsbergen and Bear Island. J. Ecol.

[b75] Symondson WOC, Harwood JD (2014). Special issue on molecular detection of trophic interactions: Unpicking the tangled bank. Mol. Ecol.

[b76] Teng J, McCann KS (2004). Dynamics of compartmented and reticulate food webs in relation to energetic flows. Am. Nat.

[b77] Thebault E, Fontaine C (2010). Stability of ecological communities and the architecture of mutualistic and trophic networks. Science.

[b78] Toft S, Wise DH (1999). Growth, development, and survival of a generalist predator fed single- and mixed-species diets of different quality. Oecologia.

[b79] Trojelsgaard K, Olesen JM (2013). Macroecology of pollination networks. Glob. Ecol. Biogeogr.

[b80] Tylianakis JM, Tscharntke T, Lewis OT (2007). Habitat modification alters the structure of tropical host–parasitoid food webs. Nature.

[b81] Várkonyi G, Roslin T (2013). Freezing cold yet diverse: dissecting a high-Arctic parasitoid community associated with Lepidoptera hosts. Can. Entomol.

[b82] Vázquez DP, Stevens RD (2004). The latitudinal gradient in niche breadth: concepts and evidence. Am. Nat.

[b83] van Veen FJF, Müller CB, Pell JK, Godfray HCJ (2008). Food web structure of three guilds of natural enemies: predators, parasitoids and pathogens of aphids. J. Anim. Ecol.

[b601] Visakorpi K, Wirta HK, Schmidt NM, Roslin T (2015). No detectable trophic cascade in a high-Arctic arthropod food web. Basic Appl. Ecol., in press.

[b84] Willig MR, Kaufman DM, Stevens RD (2003). Latitudinal gradients of biodiversity: pattern, process, scale, and synthesis. Annu. Rev. Ecol. Evol. Syst.

[b85] Wilson EO (1992). The diversity of life.

[b86] Wirta H, Hebert PDN, Kaartinen R, Prosser S, Várkonyi G, Roslin T (2014). Complementary molecular information changes our perception of food web structure. Proc. Natl Acad. Sci. USA.

[b87] Wirta H, Weingartner E, Hambäck P, Roslin T (2015). Extensive niche overlap among the dominant arthropod predators of the High Arctic. Basic Appl. Ecol.

[b88] Wise DH (2006). Cannibalism, food limitation, intraspecific competition and the regulation of spider populations. Annu. Rev. Entomol.

