# Supplementary material for: Exposing the structure of an Arctic food web
Source: Ecol Evol. 2015 Aug 24;5(17):3842–56. doi: 10.1002/ece3.1647 (PMC4567885; doi:10.1002/ece3.1647)
Supplement: Supplementary file 2 — Appendix S1. Detailed methods and additional results. [file ece30005-3842-sd2.docx]

**Exposing the structure of an Arctic food web**

**H. K. Wirta, E. J. Vesterinen, P. A. Hambäck, E. Weingartner, C. Rasmussen,** J. Reneerkens,

N. M. Schmidt, **O. Gilg, and T. Roslin**

**Appendix S1. Detailed methods and additional results**

For the current study, we combine data on trophic interactions as obtained by multiple methods, targeting predator guilds varying greatly in their way of consuming prey taxa. Birds and spiders both consume many prey individuals during their lives, while parasitoids develop and feed on a single prey (host) individual. As the per capita impact of different predator guilds on prey populations differs, and as the conversion of molecular data to quantitative information on prey use is not without challenges ([Clare 2014 and references therein](#_ENREF_2)), we focus our analyses on qualitative descriptors of predator-prey associations (with one worked-through exception below, cf. “A quantitative comparison of prey use by different bird species” below). The rationale is simple: a prey found in the gut or faecal contents of a predator offers qualitative proof of a feeding association between the two taxa. Although necessitated by practical considerations, the current restriction to a qualitative representation of the interaction network does prevent us from any analysis of relative interaction strength. Such analyses will be the attempted in future work.

**Resolving the prey species of birds**

Prey use by birds was established by identifying the remaining prey DNA from the birds’ faecal droppings collected in June-July 2013. Bird droppings were collected either when the birds were handled for ringing and for measurements ([e.g. Reneerkens *et al.* 2014](#_ENREF_9)), or from birds followed and seen defecating. All droppings were collected individually in tubes filled with 99.5% ethanol in the field, and stored in a freezer at -20 °C. Fourteen droppings for *Calidris alpina* Linnaeus were available for analyses, 43 for *Calidris alba* Pallasand 46 for *Plectrophenax nivalis* Linnaeus. Each dropping was weighted prior to DNA extraction.

For extraction, we tested several methods (including several kits, salt-extraction methods and silica-based protocols) to get the best results out of our samples (data not shown). We chose to extract total DNA individually from each pellet using ZR Fecal DNA MiniPrep Kit (product nr 131-D6010, Zymo Research Corporation), according to the instructions (manual version 1.1.2) provided with the kit. The DNA extraction and locus-specific PCR preparations were implemented in a special clean laboratory dedicated to low concentrations of DNA, such as faecal DNA. Prior to each extraction batch, the lab and the equipment were cleaned using UV light. PCR setup was done at the clean lab, but actual thermal cycling was carried out in another room to minimise the risk of contaminating the clean lab with highly concentrated DNA.

For each extract, we amplified the target locus in two separate PCR reactions. Both PCRs were carried out with the following protocol: 2 µl of the template DNA was mixed with 300 nM of each tagged locus-specific primer ([ZBJ-ArtF1c and ZBJ-ArtR2c from Zeale *et al.* 2011](#_ENREF_15)), and 6.25 µl of MyTaq RedMix (Bioline), after which the reaction was filled up to 12,5 µl with distilled water. The locus-specific PCR cycling conditions were as follows: 5 min in 96°C, then 40 cycles of 30 s in 96°C, 30 s in 50°C and 60 s in 72°C, ending with 10 min in 72°C. The locus-specific primers were tagged by special linkers to enable easy insertion of adapter in the subsequent PCR ([Clarke *et al.* 2014](#_ENREF_3)). The first PCR included a forward-tagged locus-specific forward primer, but to build a DNA library that includes both forward and reverse reads when sequenced, the second PCR included a forward-tagged reverse primer. 2 µl of the PCR product was loaded to a 2 % agarose gel and run at 95 V for 40 min.

5 µl of successful forward and reverse PCR reactions were pooled by sample. The resultant compound samples were then cleaned with 1.4 µl of Exonuclease I and 2.8 µl of FastAP Thermosensitive Alkaline Phosphatase (both from Thermo Fisher Scientific Inc., Waltham, Massachusetts, USA), by heating to 37°C for 15 min and 85°C for 15 min.

Adapter-PCR was carried out to attach IonTorrent-specific adapters and sample-specific barcodes to the PCR products of individual samples. Special, individually barcoded primers were used as follows: tagged P1-adapters 5’ CCTCTCTATGGGCAGTCGGTGATcattaagttcccatta-3’ (linker-tag in small letters) and barcoded plus tagged A-adapters 5’-CCATCTCATCCCTGCGTGTCTCCGACTCAGxxxxxxxxxxGATacgacgttgtaaaa-3’ (x’s mark the place for sample-specific barcode sequence; linker-tag in small letters). The setup for adapter-PCR was as follows: for reaction volume of 25 µl, 10 µl distilled water, 0.25 µl KAPA HiFi DNA polymerase (1U/ µl, KAPA Biosystems, Wilmington, Massachusetts, USA), 12.5 µl 2X KAPA HiFi ReadyMix and 300 nM P1 primer, 300 nMl reverse primer and 1µl purified locus-specific PCR product. The PCR cycling conditions were 3 min in 95°C, then 35 cycles of 20 s in 98°C, 15 s in 60°C and 15 s in 72°C, ending with 1 min in 72°C. To confirm adapter-PCR success, 2 µl of the adapter PCR product was loaded to a 2% agarose gel and run at 90 V for 45 min. 9 µl of the adapter PCR product was then cleaned with SPRI bead double purification ([as in Vesterinen *et al.* 2013](#_ENREF_12)), to discard un-specific PCR products longer than 400 bp and shorter than 200 bp. The procedure was as follows: 6.75 µl SPRI was added to each sample, and mixed thoroughly by vortexing. To allow the DNA to bind to the beads, the samples were then incubated 5 min at room temperature, after which the samples were placed on magnets. The supernatant (including only DNA fragments shorter than approx. 400 bp) were subsequently transferred to a new plate, 3.5 µl SPRI was added and the samples were vortexed. Again, the samples were incubated at RT for 5 min before placing them on magnet, after which the supernatant (including DNA fragments shorter than 200 bp long) were discarded. The beads, to which the targeted length PCR-products were attached to, were then washed twice with 100 µl freshly made 80% ethanol and left to dry for 20 min after the washes. 22 µl of purified and distilled water was then added to each sample, the samples were vortexed and placed on magnet, after which 20 µl of the supernatants (including the cleaned PCR product) were transferred to a new plate.

Purified adapter-PCR DNA concentrations were measured using TapeStation 2100 (Agilent Technologies, Santa Clara, California, USA), following the manufacturer’s instructions. Samples were then pooled in equimolar ratios. This combined DNA library was measured using BioAnalyzer 1600 (Agilent Technologies, Santa Clara, California, USA) and subsequently diluted into 26 pM for template preparation using Ion OneTouch™ 2 System. The template was then loaded into one 318 chip and sequenced using Ion Torrent PGM.

3,115,672 raw reads were obtained from the Ion Torrent run. After trimming of adapters and low quality parts and removing too short reads ([using FASTX-Toolkit available at http://hannonlab.cshl.edu/fastx_toolkit and USEARCH tools; Edgar 2010](#_ENREF_5)) we ended up with 546,180 final reads. These reads were combined with one Spider gut analysis dataset that was done using same methods and same sequencing platform. Overall, the combined dataset was collapsed into 10,815 unique haplotypes. Altogether, 221 OTU clusters were formed ([using USEARCH cluster_otus command with default settings; Edgar 2010](#_ENREF_5)) and these were identified to biological species by comparing each OTU to BOLD database using the following criteria:

1a 100% match to one or several species. Choosing local species.

1b At least 98% match to one or several species in one genus. Choosing local species. Choosing the best hit.

2 At least 98% match to one or several species that may be from several genera. Choosing local species. Choosing the best hit.

3 At least 98% match to one or several species that may be from several genera or on the identity of which there is no information in the database. Choosing local species. Choosing the best hit.

4 Less than 98% match to one species that is local or hits to only one species that has not been found in the study area. Likely identification, but not used in the further analysis.

5 No hits to anything biologically meaningful. Discarded.

Most of the OTUs (63) were identified with 100% match to 1 single species. Overall, 92 % of final reads were identified to species with at least confidence level 3. All the hits with at least confidence level 3 were used in the subsequent analysis.

While we here consider all the prey species found from the bird droppings as having been preyed upon by the bird species studied, some of the occurrences might result from secondary predation (in which one predator feeds on another that has recently eaten the target prey). Secondary predation cannot be separated from direct predation by identifying the prey species by DNA. As such, it is a known phenomenon worth keeping in mind not only when interpreting diet by DNA barcoding ([King *et al.* 2008](#_ENREF_6)), but also when e.g. identifying prey bones from faeces or gut contents by purely morphological means. We detected both spider and lepidopteran parasitoid DNA in bird droppings (in table S1 and below in *Intraguild predation*). While these were rare occurrences, they nonetheless show that it is possible that some of the Diptera and Lepidoptera prey DNA found in bird droppings might first have been eaten by a spider/ a lepidopteran parasitoid, and only secondly consumed by the bird. Nonetheless, on the overall patterns reported here, such rare events are likely to have a negligible impact.

**A quantitative comparison of prey use by different bird species**

While overall we focused on qualitative representations of trophic interactions among species (see above as well as Materials and Methods in the main text), we here take an in-depth and partly quantitative look at the prey use of birds. For this purpose, we use the presence or absence of a prey taxon in a given dropping as the fundamental unit of observation, still disregarding the relative abundance of sequences representing the prey in question ([for a similar approach, see Wirta *et al.* 2015](#_ENREF_14)). Based on this representation of quantitative prey use, we construct a quantitative food web of birds by package bipartite ([Dormann *et al.* 2009](#_ENREF_4)) as implemented in R ([R Core Team 2012](#_ENREF_11)).

While the abundances depicted in the web will partly reflect differences in the availability of droppings per bird species, they also reveal species-specific differences in prey use among *C. alpina* and *P. nivalis*. That the snow bunting *P. nivalis* will mainly feed on Lepidoptera, and *C. alpina* on Diptera, is evident from this representation of the data (Fig. S1). Hereby our study gives the first extensive description of the diet of Arctic arthropod-feeding birds, essential for animal ecologists with an interest in how food abundance affects birds’ responses to environmental variability (e.g. ([Reneerkens *et al.* 2011](#_ENREF_8); [Bolduc *et al.* 2013](#_ENREF_1); [Saalfeld & Lanctot 2014](#_ENREF_10))).

Figure S1. A semi-quantitative food web of prey consumed by the three most common insect-feeding birds of the study area. Blocks in the upper row represent the three bird species, and blocks in the lower row show their prey. The prey taxa are coloured by orders and numbered as in table 1 of the main text. The width of lines connecting predators and prey represents the relative abundance of the interaction in question.


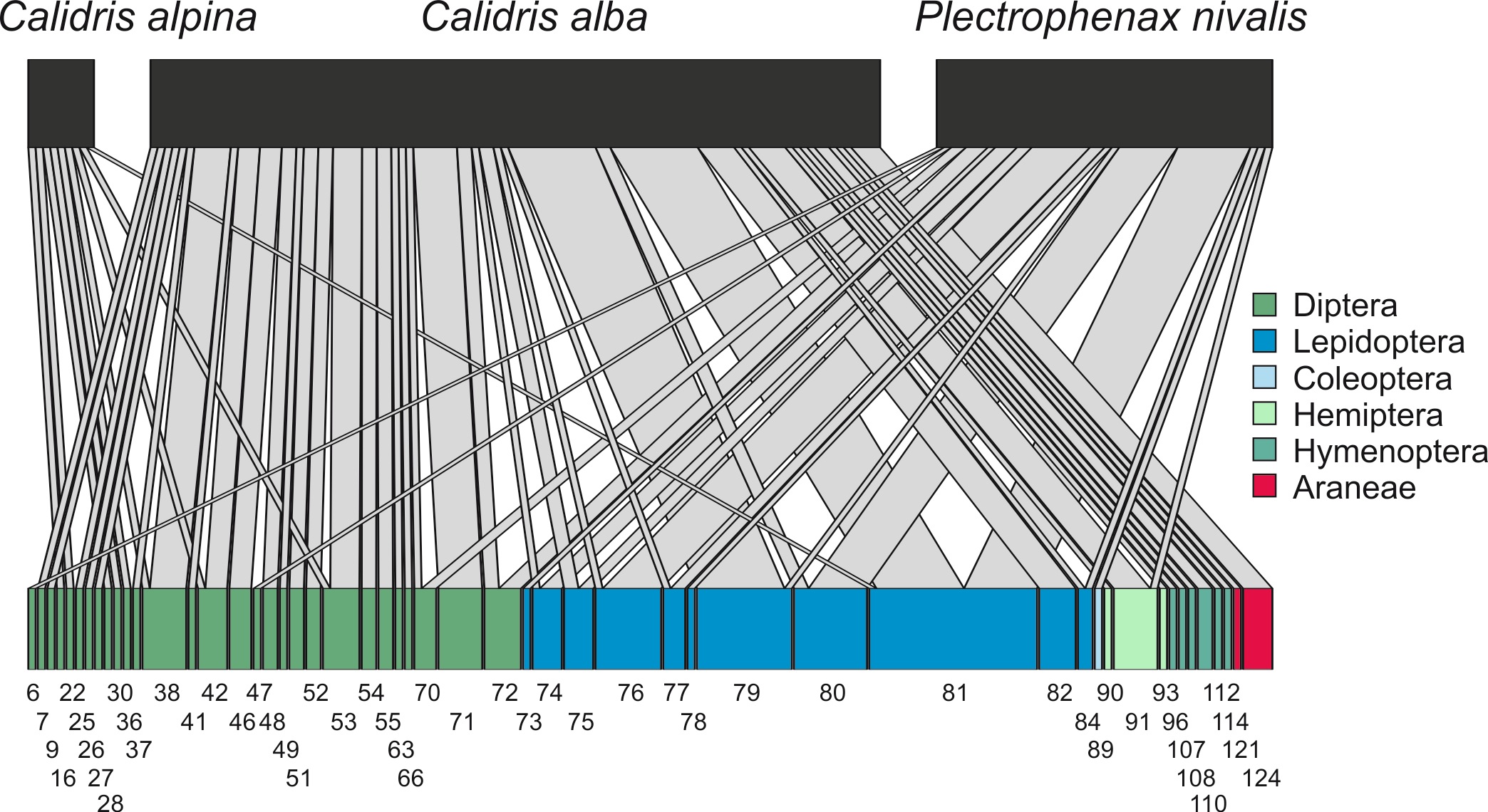


**Three methods for describing prey use by spiders**

We used three different methods for detecting prey use of the focal spiders: *Pardosa glacialis* (Thorell), *Xysticus deichmanni* Sorensen, *X. labradorensis* Keyserling, *Emblyna borealis* (O. Pickard-Cambridge) and *Erigone arctica* White. By each method, we identified the prey remains from the gut of the spiders based on DNA barcoding. For Method 1, we used selective amplification of prey remains of individual spiders by primers specific to Diptera and Lepidoptera, followed by direct Sanger sequencing ([Wirta *et al.* 2015](#_ENREF_14)), for Method 2 we used pooled samples, which were amplified with Diptera and Lepidoptera specific primers and then sequenced with high throughput sequencing (below), and for Method 3, we amplified pooled samples with general arthropod primers followed by high throughput sequencing (below). For birds, we used only Method 3 (as described above), and for Lepidopteran parasitoids, we used a combination of taxon (order- or family-) specific primers and Sanger sequencing as well as rearing of host larvae ([Wirta *et al.* 2014](#_ENREF_13)).

As discussed by ([Wirta *et al.* 2015](#_ENREF_14)), the two *Xysticus* species occurring in our study region can be distinguished by DNA barcode. For one of the methods used here, the halved cephalothoraxes of *Xysticus* samples were pooled directly after cutting for DNA extraction, and could therefore not be identified to species a posteriori. As Wirta *et al.* ([2015](#_ENREF_14)) found no differentiation in prey use between the two *Xysticus* species, *X. deichmanni* and *X. labradorensis*, we here combine them as a compound taxon *Xysticus* spp.

The samples used in all the three methods were collected individually during June-August 2012, and stored in a freezer, as described in Wirta *et al.* ([2015](#_ENREF_14)). In total, we used 120 individuals of *P. glacialis* and 120 of *Xysticus* spp. The spiders were halved and DNA extracted singly from one of these half (using the same DNA extracts for Methods 1 and 3), then amplified and sequenced with Diptera-Lepidoptera specific primers for Method 1 ([Wirta *et al.* 2015](#_ENREF_14)). The other halves were used for Method 2 (below). Individuals of *E. borealis* (120 individuals) and *E. arctica* (ten individuals) were collected, preserved and processed in the same way and analysed with the method 3 together with samples of *P. glacialis* and *Xysticus* spp. The small-bodied *E. borealis* and *E. arctica* were also cut open with sterile equipment to expose the gut contents similar to the other species, but both halves were used in the same DNA extraction.

*Extracting, amplifying and sequencing pooled samples with Diptera-Lepidoptera specific primers*

The extraction was carried out in a clean laboratory dedicated to low concentrations of DNA (cf. above under *Extracting, amplifying and sequencing pooled samples with Diptera-Lepidoptera specific primers*). The halves of the spider individuals were combined as pools of 9-15 individuals per taxon and sex. Each pool was mixed with 2.5 mm zirconia/silica beads (Biospec) and homogenized in a Mini-beadbeater with 540 with 540 a Mini-beath 540 0 40 L. The tubes were incubated in 56beater beater a beads (Biospec) and homogenized in a Mini-beadbea per pool) and extracted according to the protocol using QIAamp (QIAGEN). The extracts were diluted x10 and stored in -20 °C.

These pooled extracts were amplified with the Diptera-Lepidoptera-specific primers with a 6 bp ”barcode” tag at the 5’ end of the primer. Each PCR thus had a unique primer combination. Two PCR reactions were performed per extract and the two different replicates were prepared on different plates later marked with MID-primer tags (see below): The reactions were carried out in a total volume of 20 µl including; 2.0 µl 10x buffer, 0.8 µl MgCl_2_, 2.0 µl BSA, 0.4 µl dNTP (200 μM of each dNTP ), 4.0 µl of each primer (2 µM final concentration each), 0.2 µl HotStarTaq (Qiagen), 2.0 µl DNA template and water. The thermal cycling profile started with a denaturating step of 5 min in 95°C, followed by 45 cycles with denaturating for 30 s. in 94°C, annealing for 30 s. in 52°C, extension for 1 min in 65°C, and finally an extension step for 8 min in 65°C. Blanks were included among the PCRs. The amplified products were checked on a “long stretch“ 1.8% agarose gel. No blanks appeared on the gel. The PCR products were normalized using SequalPrep^TM^ Normalization Plate Kit (Life Technologies) following the protocol with 17 µl of each product and 17 µl binding buffer loaded in the wells. The normalized PCR products were eluted in 20 µl elution buffer, pooled and concentrated using Vivaspinn filters 30K MWCO (Sartorius) and purified using the MinElute purification kit (QIAGEN). The concentrations of the pools were estimated on a gel against a 100 bp Plus DNA Ladder (Fermentas) in different concentrations. A rapid library was prepared for each pool and adaptors were ligated according to the Rapid Library Preparation Method Manual (Roche 454 Sequencing) with the following exceptions: The first step of DNA fragmentation by nebulization was ignored. In the next step (3.2) only the phosphorylation and A-tailing were performed, thus the DNA samples was mixed with EB buffer, RL buffer, RL ATP and RL PNK. RL MID7 and MID8 Adaptors were added in step 3.4. Instead of the small fragment removal suggested in step 3.5, the libraries were purified using MinElute columns (Qiagen) with the elution volume of 25 µl. The libraries were loaded to a 0.8% agarose gel in 1 X TBE buffer together with 100 bp Plus DNA Ladder (Fermentas) and run for 1 hour at 80 V. The bands of approximately 350 bp were cut from the gel and purified with the QIAQuick Gel Extraction kit (Qiagen). The protocol ”QIAquick Gel Extraction kit using a Microcentrifuge” was followed with the exceptions that the agarose was melted only using vortexing, incubating time in step 10 was 2-5 minutes before spinning and in the PE dry spin step the tubes were rotated and spun additionally 1 min. The libraries were eluted in 50 µl EB buffer. The Library Quality Assessment was done using the Agilent Bioanalyzer, following the protocol ”Agilent High Sensitivity DNA Kit Guide”. Library Quantitation was done using the TBS 380 Fluorometer.

The emulsion PCR preparation was done according to the protocol ”emPCR Amplification Method Manual - Lib-L” (Roche). During the preparation of the Mock Mix and Pre-Emulsion, the Amp Mix was prepared according to table 1B in the protocol. The DNA library was calculated to be amplified 0.7 molecules/bead. The amplification reaction was run for 35 cycles.

The libraries were sequenced on a Roche GS Junior (454 Sequencing technique) following the Sequencing Method Manual, March 2012. The whole run (which also included some separate Swedish spider samples) resulted in 125,565 sequences passing the filter. The sequences were sorted per primer combination and adaptors and tags were removed using a perl script by Johan Nylander (BILS, SciLife Laboratories, Stockholm, Sweden). 47,961 sequences (only Greenland sequences) were further trimmed using Tagcleaner available at <http://edwards.sdsu.edu/cgi-bin/tagcleaner/tc.cgi>. Primers were removed and only sequences shorter than 280 were discarded. Altogether, 47,844 trimmed reads were collapsed into 16,072 unique haplotypes and further clustered into 238 OTUs. OTU clustering and identification to biological species was done as explained above (see “Resolving the prey species of birds”).

*Extracting, amplifying and sequencing pooled samples with general primers*

As a third method to examine prey use by spiders, we adopted the method of Pinõl *et al.* ([2014](#_ENREF_7)). For this purpose, we used primers amplifying all arthropods, and included both the three spider taxa analysed with the first and second method and two additional species, *E. arctica* (Linyphiidae; with only mature specimen selected) and *E. borealis* (Dictynidae; with also juveniles included).

For DNA extraction, the cephalothorax and the abdomen of the small bodied species *E. borealis* and *E. arctica* were halved to expose the gut contents, and all body parts included. DNA was extracted as in Wirta *et al.* ([2015](#_ENREF_14)). For *P. glacialis* and *Xysticus* spp., we used the singly-extracted DNAs from Wirta *et al.* ([2015](#_ENREF_14)).

The DNA contents of extracts were measured twice by NanoDrop (Thermo Fisher Scientific Inc., Waltham, Massachusetts, USA). In cases where different concentrations were recorded, we adopted the average of the two measurements as our final reading. The DNA extracts were then combined into pooled samples of three to five specimens, with all the samples of a pool chosen to represent the same sex, age class (mature vs juvenile) and time period of summer (see ([Wirta *et al.* 2015](#_ENREF_14))). From each DNA extract (representing a single specimen), we added 500 ng of DNA to the pooled sample. These pools were then amplified three times for the target locus with the following PCR protocol: for the reaction volume of 15 µl, 2 µl of the template DNA was used with 8.65 µl distilled water, 0.15 µl HotStarTaq DNA polymerase (Qiagen), 1.5 µl MgCl_2_ (25 mM) and 1.5 µl 10x buffer (MgCl_2_ and buffer provided with HotStarTaq), 0.6 µl dNTP mix (10mM of each base) together with 2 µM of each primer. The locus-specific primers were tagged by linker-tags to enable easy insertion of adapter in the subsequent PCR (modified from ([Clarke *et al.* 2014](#_ENREF_3))). The locus-specific PCR cycling conditions were as follows: 15 min in 95°C, then 35 cycles of 30 s in 95°C, 30 s in 50°C and 60 s in 72°C, ending with 10 min in 72°C. For samples with a faint band in the first PCR, 4 µl of the template DNA was used with 6.65 µl distilled water in the latter two PCRs and otherwise the protocol remained the same.

4 µl of the PCR product was loaded to a 1% agarose gel and run with 95 V for 40 min. 5 µl of each PCR was taken into combined samples. These were cleaned with 0.75 µl of Exonuclease I and 3 µl of FastAP Thermosensitive Alkaline Phosphatase (both from Thermo Fisher Scientific Inc., Waltham, Massachusetts, USA), with heating to 37°C for 30 min and 85°C for 15 min.

The resultant combined and cleaned locus-specific PCR products were used for adapter-PCR, attaching IonTorrent-specific adapters and the sample-specific barcodes into the samples. Adapter-PCR was carried out exactly as earlier explained (see Chapter “Resolving birds’ prey species”), except for the PCR setup which was slightly different: for reaction volume of 12.5 µl, 7.625 µl distilled water, 0.25 µl KAPA HiFi DNA polymerase (1U/ µl, KAPA Biosystems, Wilmington, Massachusetts, USA), 2.5 µl 5X KAPA HiFi buffer (Fidelity) and 0.375 µl 10mM KAPA dNTP Mix (both buffer and dNTP mix provided with the KAPA HiFi DNA polymerase), 0.3 µM P1 primer, 0.3 µM A-primer and 1µl purified locus-specific PCR product. The PCR cycling conditions were 3 min in 95°C, then 35 cycles of 20 s in 98°C, 15 s in 60°C and 15 s in 72°C, ending with 1 min in 72°C.

2 µl of the adapter PCR product was loaded to a 2% agarose gel and run with 90 V for 45 min. 9 µl of the adapter PCR product was then cleaned with SPRI bead double purification exactly the same way as earlier (see Chapter “Resolving the prey species of birds”). The purified adapter-PCR DNA concentrations were measured using Qubit Fluorometer (Life Technologies/ Thermo Fisher Scientific Inc., Waltham, Massachusetts, USA), following the manufacturer’s instructions. 45 ng of each sample were pooled into one DNA library. This combined DNA library was measured using BioAnalyzer (Agilent Technologies, Santa Clara, California, USA) and diluted into 26 pM for template preparation using Ion OneTouch™ 2 System. The template was then loaded into one 318 chip and sequenced using Ion Torrent PGM.

7,446,579 raw reads were obtained from the Ion Torrent run. After trimming of adapters and low quality parts and removing too short reads we ended up with 1,143,799 final reads. These reads were combined with the Bird Prey analysis dataset and analysed as explained above (see “Resolving the prey species of birds”). The proportion of reads originating from predator itself by each spider species was as follows: *Emblyna borealis* 94.1 %, *Erigone arctica* 17.0 %, *Pardosa glacialis* 95 %, *Xysticus deichmanni* 99.9 % and *X. labradorensis* 16.1 %, while the number of actual prey count for the spiders was 17, 4, 3, 5 and 5, respectively.

*Comparison of prey use as resolved by different methods*

To assess whether different methods yielded the same impression of prey use by individual spider taxa, we focused on Dipteran and Lepidopteran prey use by the two predator taxa examined by all the three methods, *P. glacialis* and *Xysticus* spp. Qualitative food webs were generated for each taxon with package bipartite ([Dormann *et al.* 2009](#_ENREF_4)).

As revealed by the two food webs shown in the Fig. S2, a similar prey composition and diet width was detected by Methods 1 and 2 as targeting Dipteran and Lepidopteran prey. The few differences in the prey composition encountered between these methods can likely be attributed to the fact that different halves of the body were used with different methods, and that the contents of DNA extracts can thus differ between the single and pooled samples used. As a result, the two halves of the spider body could simply exhibit some differences in their contents of prey remains. The DNA of different prey may also be differently extracted and amplified from the more complex pooled samples than from individual samples. On the other hand, the third method of general arthropod primers and high throughput sequencing with Ion Torrent yielded very few prey items, as most of the sequences obtained belonged to the predator themselves (see above). Thus, this last method seems less promising than suggested by the original authors ([Piñol *et al.* 2014](#_ENREF_7)).

Figure S2. Qualitative food webs for A) *P. glacialis* and B) *Xysticus* spp., with prey resolved by three methods: 1) Method 1: selective amplification of Diptera and Lepidoptera from individual samples, followed by Sanger sequencing, 2) Method 2: selective amplification of Diptera and Lepidoptera from pooled samples, followed by high through put sequencing and 3) Method 3: amplification with general arthropod primers of pooled samples, followed by high through put sequencing. The blocks on the upper row represent different methods used, and the blocks in the lower row represent different prey species. Thus, should each method detect each link, we would see links radiating from each bock on the upper row to each block on the lower – barring effects of sample size. Note that the width of the blocks is only related to the number of links detected, not to sample sizes. The prey species are numbered as in Table 1 in the main text. The colour of each prey block identifies family, with dipteran families shown in red and purple, and lepidopteran families indicated by different shades of blue. Overall, links radiating from Method 1 and 2 appear remarkably similar, whereas links from Method 3 are constrained by the low number of prey sequences detected by this method (see section *Comparison of prey use as resolved by different methods*).


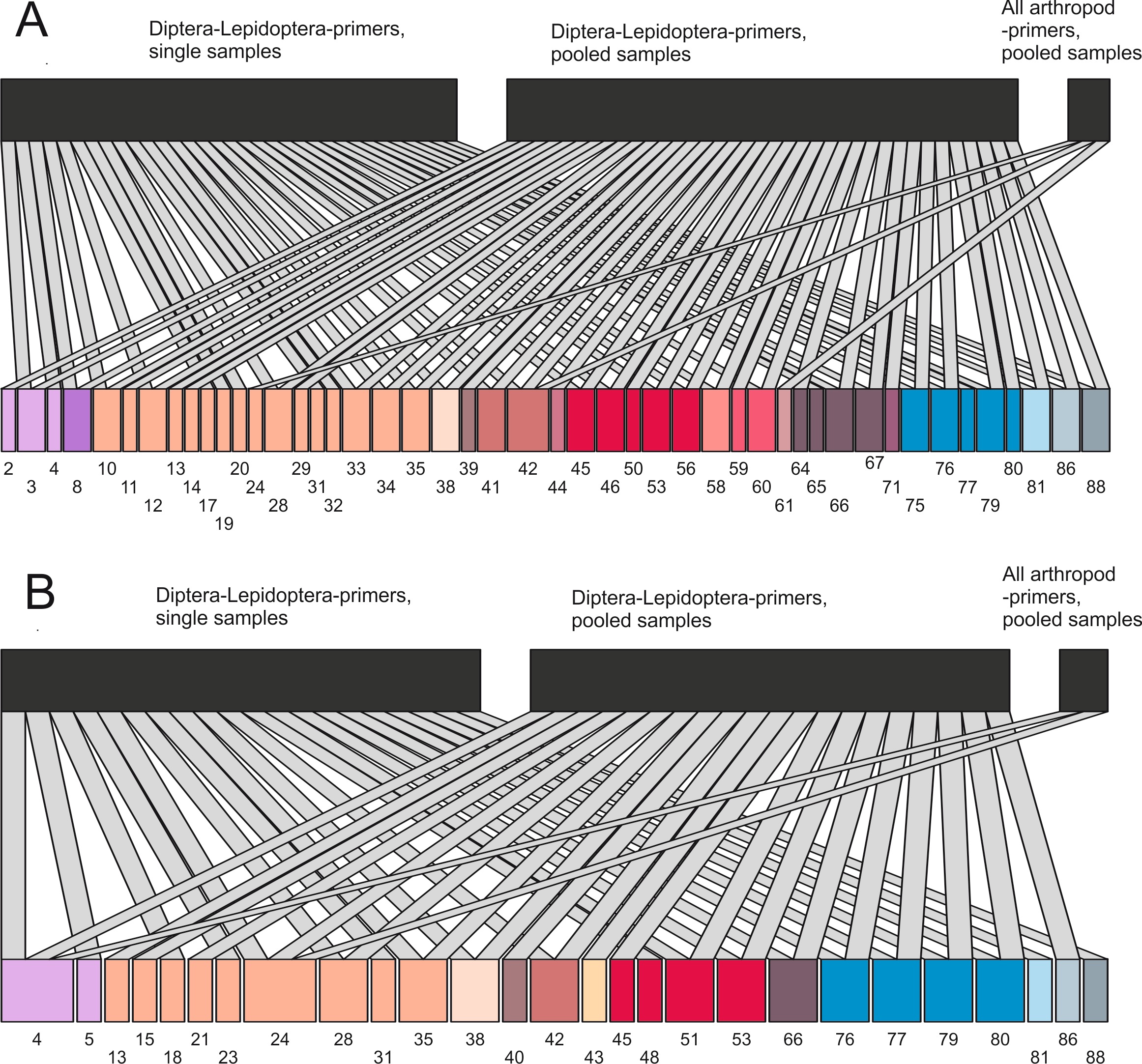


**Intra-guild predation is poorly resolved – but adds complexity to the web**

For completeness, all trophic links to all prey orders are reported in supplementary table S1and part of them present intra-guild predation among different predator groups (Fig. S3). While resolved poorly in the current study, we believe such links among predators to further increase the complexity of the Arctic food web. Given our focus on trophic links involving dipteran and lepidopteran prey, only a subset of the methods employed will yield any information on intra-guild predation among different predator groups. Such predation events could be detected by two of our methods: for birds, we used general arthropod primers to detect the prey contents of droppings, and for spiders, we used general arthropod primers in the context of Method 3. Figure S3 illustrates the trophic links detected among birds, spiders and parasitoids of Lepidoptera by these specific means. Nonetheless, our current techniques offer only a very first impression of the *potential* for such links, and fall far short of revealing their full complexity.

Figure S3. Qualitative food web of predator-predator interactions among birds (top), spiders (middle) and parasitoids of Lepidoptera (bottom). A line connecting two species indicates that the upper species has consumed the lower one.* For simplicity, only predator species for which predator-predator interactions were detected are shown. The species are numbered as in the table 1 in the main text.

* Here two *Xysticus* species are shown separately, as with the method 3 used in the current study, the molecular markers used allowed us to detect the consumption of *Xysticus deichmanni* (125a) by *X.* *labradorensis* (125b).


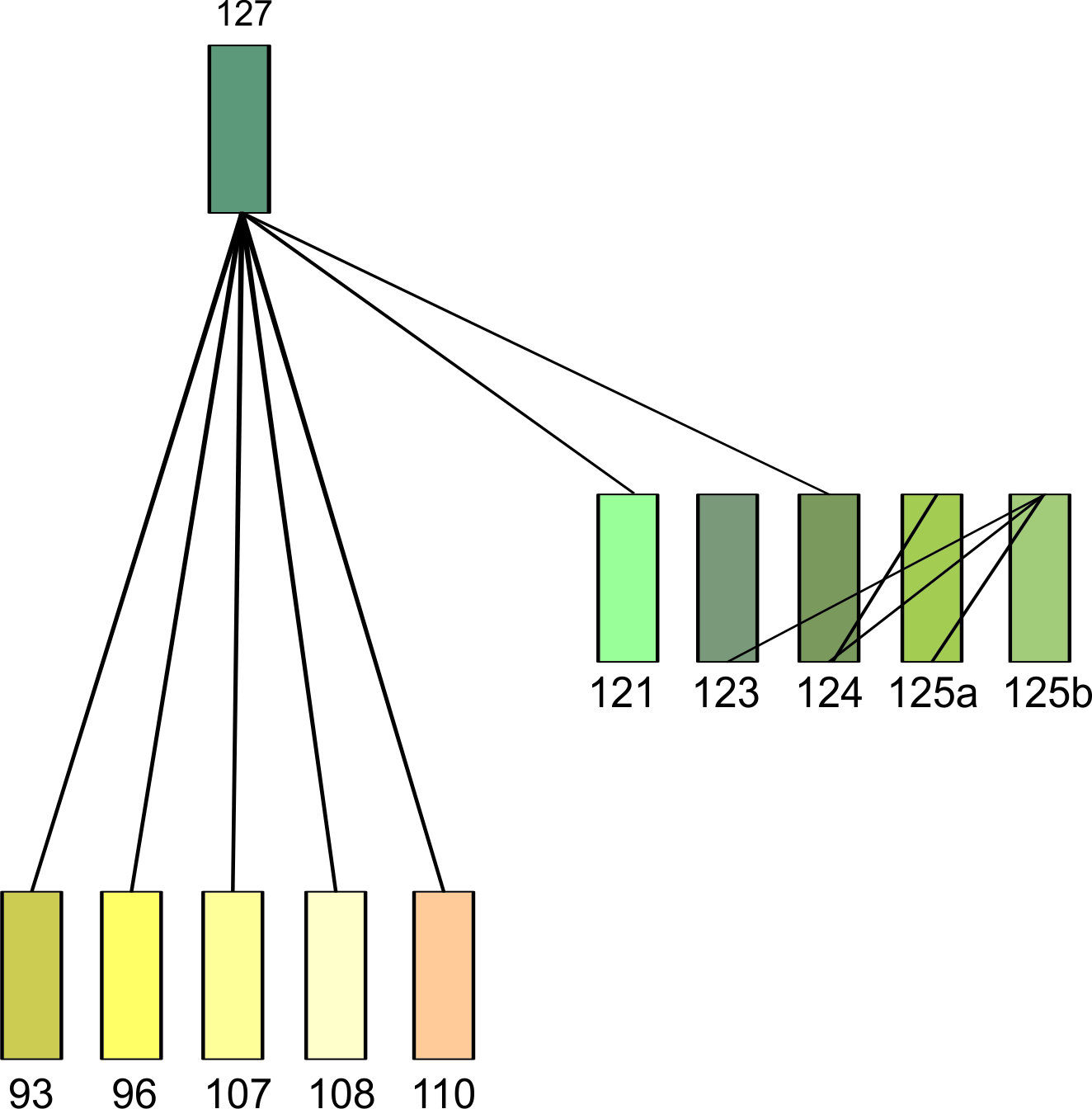


**References for Appendix S1**

Bolduc, E., Casajus, N., Legagneux, P., McKinnon, L., Gilchrist, H.G., Leung, M., Morrison, R.I.G., Reid, D., Smith, P.A., Buddle, C.M. & Bêty, J. (2013) Terrestrial arthropod abundance and phenology in the Canadian Arctic: modelling resource availability for Arctic-nesting insectivorous birds. *The Canadian Entomologist,* 145**,** 155–170.

Clare, E.L. (2014) Molecular detection of trophic interactions: emerging trends, distinct advantages, significant considerations and conservation applications. *Evolutionary Applications,* 7**,** 1144-1157.

Clarke, L.J., Czechowski, P., Soubrier, J., Stevens, M.I. & Cooper, A. (2014) Modular tagging of amplicons using a single PCR for high-throughput sequencing. *Molecular Ecology Resources,* 14**,** 117–121.

Dormann, C.F., Fründ, J., Blüthgen, N. & Gruber, B. (2009) Indices, graphs and null models: Analyzing bipartite ecological networks. *The Open Ecology Journal,* 2**,** 7-24.

Edgar, R.C. (2010) Search and clustering orders of magnitude faster than BLAST. *Bioinformatics,* 26**,** 2460-2461.

King, R.A., Read, D.S., Traugott, M. & Symondson, W.O.C. (2008) Molecular analysis of predation: a review of best practice for DNA-based approaches. *Molecular Ecology,* 17**,** 947–963.

Piñol, J., San Andrés, V., Clare, E.L. & Symondson, W.O.C. (2014) A pragmatic approach to the analysis of diets of generalist predators: the use of next-generation sequencing with no blocking primers. *Molecular Ecology Resources,* 14**,** 18-26.

Reneerkens, J., Grond, K., Schekkerman, H., Tulp, I. & Piersma, T. (2011) Do uniparental sanderlings *Calidris alba* increase egg heat input to compensate for low nest attentiveness? *PLoS ONE,* 6**,** e16834.

Reneerkens, J., van Veelen, P., van der Velde, M., Luttikhuizen, P. & Piersma, T. (2014) Within-population variation in mating system and parental care patterns in the Sanderling (*Calidris alba*) in northeast Greenland. *The Auk: Ornithological Advances,* 131**,** 235–247.

Saalfeld, S.T. & Lanctot, R.B. (2014) Conservative and opportunistic settlement strategies in Arctic-breeding shorebirds. *The Auk: Ornithological Advances,* 312**,** 212-234.

Team, R.C. (2012) R: a language and environment for statistical computing. Vienna, Austria.

Vesterinen, E.J., Lilley, T., Laine, V.N. & Wahlberg, N. (2013) Next Generation Sequencing of Fecal DNA Reveals the Dietary Diversity of the Widespread Insectivorous Predator Daubenton’s Bat (*Myotis daubentonii*) in Southwestern Finland. *PLoS ONE,* 8**,** e82168.

Wirta, H., Hebert, P.D.N., Kaartinen, R., Prosser, S., Várkonyi, G. & Roslin, T. (2014) Complementary molecular information changes our perception of food web structure. *Proceedings of the National Academy of Sciences of the United States of America,* 111**,** 1885-1890.

Wirta, H., Weingartner, E., Hambäck, P. & Roslin, T. (2015) Extensive niche overlap among the dominant arthropod predators of the High Arctic. *Basic and Applied Ecology,* 16**,** 86-92.

Zeale, M.R.K., Butlin, R.K., Barker, G.L.A., Lees, D.C. & Jones, G. (2011) Taxon-specific PCR for DNA barcoding arthropod prey in bat faeces. *Molecular Ecology Resources,* 11**,** 236-244.
