# Supplementary material for: Exposing the structure of an Arctic food web
Source: Ecol Evol. 2015 Aug 24;5(17):3842–56. doi: 10.1002/ece3.1647 (PMC4567885; doi:10.1002/ece3.1647)
Supplement: Supplementary file 3 — Appendix S2. Further components of the Zackenberg interaction web. [file ece30005-3842-sd3.docx]

**Exposing the structure of an Arctic food web**

**H. K. Wirta, E. J. Vesterinen, P. A. Hambäck, E. Weingartner, C. Rasmussen,** J. Reneerkens,

N. M. Schmidt, **O. Gilg, and T. Roslin**

**Appendix S2. Further components of the Zackenberg interaction web**

To provide further support for the notion that complexity is a general feature of the Zackenberg interaction web – rather than a characteristic of a given guild, a given trophic level or some given type of interaction – we here describe two local interaction networks beyond the predator-prey food webs discussed in the main paper: As an example of a mutualistic interaction web, we show the local pollinator-plant web reconstructed from the data of ([Rasmussen *et al.* 2013](#_ENREF_5)). As an example of an antagonistic food web, we show a local herbivore-plant web based on data from ([Roslin *et al.* 2013](#_ENREF_6)). Both of these interaction webs were reconstructed, and their potential compartmentalization assessed, by the package bipartite ([Dormann *et al.* 2009](#_ENREF_1)) as implemented in R ([R Core Team 2012](#_ENREF_4)). For the specific methods used to collect the data for the respective trophic interactions, we refer the reader to the original source papers (pollinators and plants, Fig. S4 A; ([Rasmussen *et al.* 2013](#_ENREF_5)), herbivores and plants, Fig. S4 B ([Roslin *et al.* 2013](#_ENREF_6))) and to the Materials and methods section in the main paper and Text S1 of the current study.

In the current context, it is important to notice that each of the networks presented in this Appendix was reconstructed by different techniques. Given this variation, we have refrained from presenting any links between them – as this set of links would be conditional on the set of methods used to reconstruct them. Instead, we present the different communities separately, seeking for general patterns in their structure, and for traits either conforming to or departing from the patterns described in the main paper.

As main findings, both the mutualistic and the antagonistic interaction webs among plants and arthropods (Fig. S4 A and B) show a densely-linked structure. Moreover, they both consist of a single compartment, implying that each species in the web is connected to all other species through shared pollinators, herbivores and/or host plants. In all of these features, they match the predator-prey webs described in the main text. Overall, we believe that these representations all show joint support for the notion that our target web is characterized by dense links – across biotic interactions of many kinds.

What our study does not adequately resolve are the links between interactions of different types. It is well evident that the trophic food web of predator-prey interactions (cf. main paper) is intricately linked with the other types of interaction webs presented in this Appendix. Of the prey taxa targeted in the main paper, Diptera form the main pollinators in this community ([Elberling & Olesen 1999](#_ENREF_2); [Rasmussen *et al.* 2013](#_ENREF_5)), and Lepidoptera are pollinators as adults but herbivores as larvae ([Roslin *et al.* 2013](#_ENREF_6)). Linking the separate modules into a regional interaction ‘metaweb’ ([cf. Pocock, Evans & Memmott 2012](#_ENREF_3)) is thus our ultimate objective, and the subject of ongoing work.

Figure S4. Local interaction webs for supplementary interaction types at Zackenberg: A) pollinators and the plants that they visit ([Rasmussen *et al.* 2013](#_ENREF_5)) and B) lepidopteran herbivores and the plants that they consume ([Roslin *et al.* 2013](#_ENREF_6)). The species are numbered as in the table S3 below.


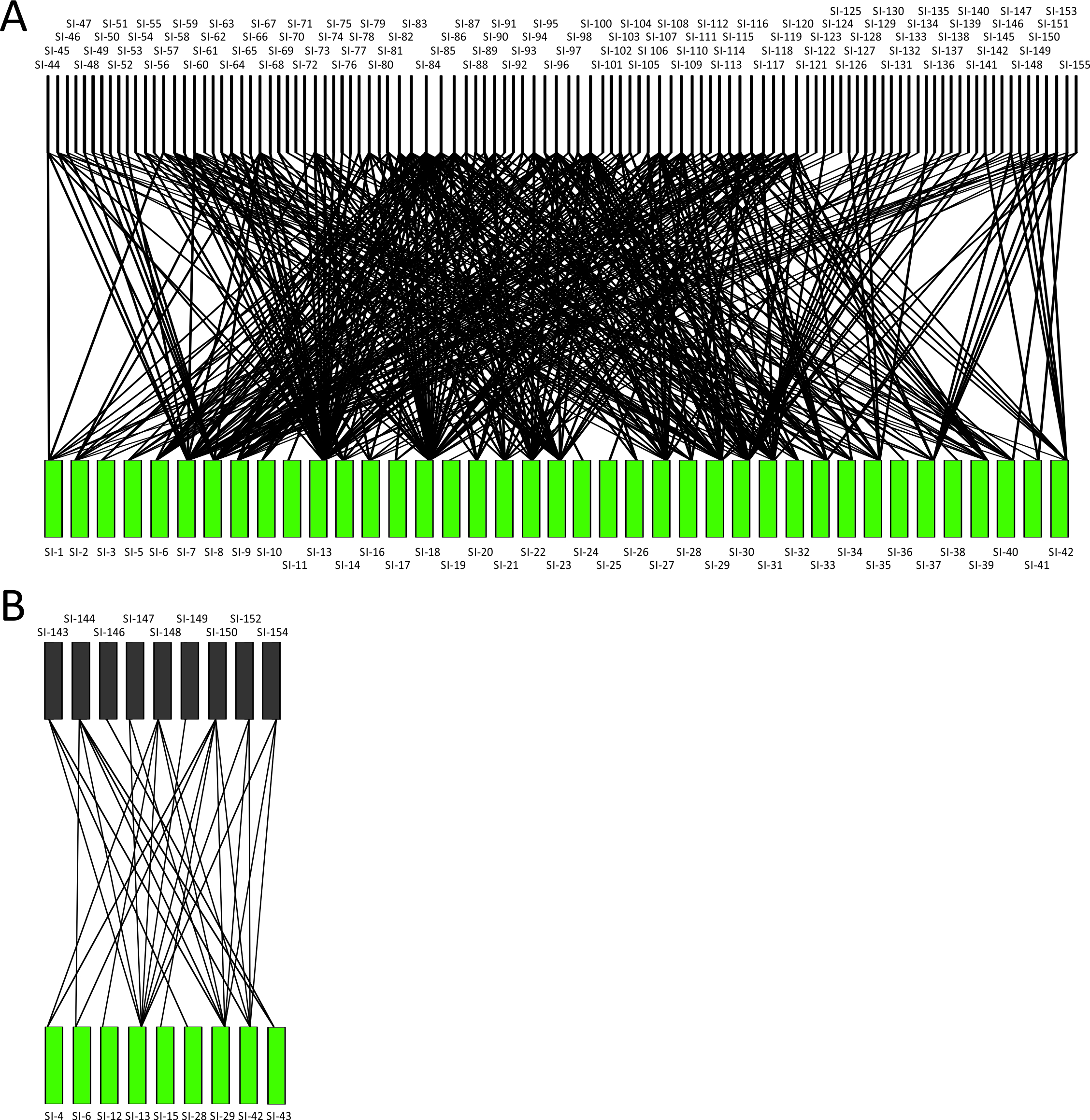


Table S2. Plant, pollinator and herbivore species forming the mutualistic interaction web of pollinators and plants in Fig S4 A ([Rasmussen *et al.* 2013](#_ENREF_5)) and the antagonistic food web of lepidopteran herbivores and plants in Fig S4 B ([Roslin *et al.* 2013](#_ENREF_6)).

|  | Plant |  | Pollinator/ herbivore | | |
| --- | --- | --- | --- | --- | --- |
| # | species | # | order | family | species |
| SI-1 | *Arenaria pseudofrigida* | SI-44 | Diptera | Agromyzidae | *Phytomyza fuscula* |
| SI-2 | *Armeria scabra* | SI-45 |  | Anthomyiidae | *Delia echinata* |
| SI-3 | *Arnica angustifolia* | SI-46 |  |  | *Fucellia pictipennis* |
| SI-4 | *Betula nana* | SI-47 |  |  | *Paradelia arctica* |
| SI-5 | *Cardamine pratensis* | SI-48 |  |  | *Pegomya icterica* |
| SI-6 | *Cassiope tetragona* | SI-49 |  |  | *Zaphne divisa* |
| SI-7 | *Cerastium arcticum* | SI-50 |  |  | *Zaphne frontata* |
| SI-8 | *Chamaenerion latifolium* | SI-51 |  |  | *Zaphne occidentalis* |
| SI-9 | *Cochlearia groenlandica* | SI-52 |  |  | *Zaphne tundrica* |
| SI-10 | *Draba arctica* | SI-53 |  | Calliphoridae | *Cynomya mortuorum* |
| SI-11 | *Draba lactea* | SI-54 |  | Ceratopogonidae | *Brachypogon* spp. |
| SI-12 | *Draba* spp. | SI-55 |  |  | *Culicoides* sp. |
| SI-13 | *Dryas octopetala* | SI-56 |  | Chironomidae | *Chironomus hyperboreus* |
| SI-14 | *Erigeron compositus* | SI-57 |  |  | *Cricotopus magus* |
| SI-15 | *Eriophorium triste* | SI-58 |  |  | *Cricotopus* spp. |
| SI-16 | *Lesquerella arctica* | SI-59 |  |  | *Limnophyes asquamatus* |
| SI-17 | *Melandrium triflorum* | SI-60 |  |  | *Limnophyes brachytomus* |
| SI-18 | *Papaver radicatum* | SI-61 |  |  | *Limnophyes* cf. *natalensis* |
| SI-19 | *Pedicularis flammea* | SI-62 |  |  | *Limnophyes* cf. *ninae* |
| SI-20 | *Pedicularis hirsuta* | SI-63 |  |  | *Limnophyes ninae* |
| SI-21 | *Polygonum viviparum* | SI-64 |  |  | *Limnophyes* spp. |
| SI-22 | *Potentilla hyparctica* | SI-65 |  |  | *Metriocnemus* spp. |
| SI-23 | *Potentilla rubricaulis* | SI-66 |  |  | *Orthocladius* spp. |
| SI-24 | *Pyrola grandiflora* | SI-67 |  |  | *Paraphaenocladius impensus* |
| SI-25 | *Ranunculus hyperboreus* | SI-68 |  |  | *Procladius paragretis* |
| SI-26 | *Ranunculus pygmaeus* | SI-69 |  |  | *Psectrocladius limbatellus* |
| SI-27 | *Ranunculus sulphureus* | SI-70 |  |  | *Pseudosmittia* cf. *nanseni* |
| SI-28 | *Rhododendron lapponicum* | SI-71 |  |  | *Pseudosmittia nanseni* |
| SI-29 | *Salix arctica* | SI-72 |  |  | *Pseudosmittia* spp. |
| SI-30 | *Saxifraga caespitosa* | SI-73 |  |  | *Rheocricotopus chapmani* |
| SI-31 | *Saxifraga cernua* | SI-74 |  |  | *Smittia* sp. 17 |
| SI-32 | *Saxifraga hirculus* | SI-75 |  |  | *Smittia* sp. 2 |
| SI-33 | *Saxifraga hyperborea* | SI-76 |  |  | *Smittia* sp. aff. *edwardsi* |
| SI-34 | *Saxifraga nivalis* | SI-77 |  |  | *Smittia* sp. x |
| SI-35 | *Saxifraga oppositifolia* | SI-78 |  |  | *Smittia* sp. y |
| SI-36 | *Saxifraga rivularis* | SI-79 |  |  | *Smittia* spp. |
| SI-37 | *Silene acaulis* | SI-80 |  |  | *Tanytarsus* sp. 1 |
| SI-38 | *Stellaria humifusa* | SI-81 |  | Culicidae | *Aedes impiger* |
| SI-39 | *Stellaria longipes* | SI-82 |  |  | *Aedes nigripes* |
| SI-40 | *Taraxacum arcticum* | SI-83 |  | Empididae | *Rhamphomyia filicauda* |
| SI-41 | *Taraxacum phymatocarpum* | SI-84 |  |  | *Rhamphomyia nigrita* |
| SI-42 | *Vaccinium uliginosum* | SI-85 |  | Muscidae | *Drymeia groenlandica* |
| SI-43 | grass | SI-86 |  |  | *Drymeia segnis* |
|  |  | SI-87 |  |  | *Limnophora groenlandica* |
|  |  | SI-88 |  |  | *Lophosceles minimus* |
|  |  | SI-89 |  |  | *Phaonia bidentata* |
|  |  | SI-90 |  |  | *Spilogona almqvistii* |
|  |  | SI-91 |  |  | *Spilogona arcticola* |
|  |  | SI-92 |  |  | *Spilogona deflorata* |
|  |  | SI-93 |  |  | *Spilogona denudata* |
|  |  | SI-94 |  |  | *Spilogona dorsata* |
|  |  | SI-95 |  |  | *Spilogona malaisei* |
|  |  | SI-96 |  |  | *Spilogona megastoma* |
|  |  | SI-97 |  |  | *Spilogona micans* |
|  |  | SI-98 |  |  | *Spilogona obsoleta* |
|  |  | SI-99 |  |  | *Spilogona sanctipauli* |
|  |  | SI-100 |  |  | *Spilogona tendipes* |
|  |  | SI-101 |  |  | *Spilogona tornensis* |
|  |  | SI-102 |  |  | *Spilogona zaitzevi* |
|  |  | SI-103 |  | Phoridae | *Megaselia arcticae* |
|  |  | SI-104 |  | Piophilidae | *Lasiopiophila pilosa* |
|  |  | SI-105 |  | Scathophagidae | *Gonarcticus arcticus* |
|  |  | SI-106 |  |  | *Scathophaga furcata* |
|  |  | SI-107 |  |  | *Scathophaga nigripalpis* |
|  |  | SI-108 |  | Sciaridae | *Bradysia* spp. |
|  |  | SI-109 |  |  | *Lycoriella* sp. 1 |
|  |  | SI-110 |  | Syrphidae | *Eupeodes punctifer* |
|  |  | SI-111 |  |  | *Eupeodes rufipunctatus* |
|  |  | SI-112 |  |  | *Helophilus groenlandicus* |
|  |  | SI-113 |  |  | *Helophilus lapponicus* |
|  |  | SI-114 |  |  | *Parasyrphus tarsatus* |
|  |  | SI-115 |  |  | *Platycheirus carinatus* |
|  |  | SI-116 |  |  | *Platycheirus lundbecki* |
|  |  | SI-117 |  |  | *Syrphus torvus* |
|  |  | SI-118 |  | Tachinidae | *Peleteria aenea* |
|  |  | SI-119 | Hemiptera | Lygaeidae | *Nysius groenlandicus* |
|  |  | SI-120 | Hymenoptera | Ichneumonidae | *Atractodes alpestris* |
|  |  | SI-121 |  |  | *Atractodes aterrimus* |
|  |  | SI-122 |  |  | *Atractodes* sp. 1 |
|  |  | SI-123 |  |  | *Buathra laborator* |
|  |  | SI-124 |  |  | *Gelis maesticolor* |
|  |  | SI-125 |  |  | *Gelis* sp. 1 |
|  |  | SI-126 |  |  | *Neurateles* sp. 1 |
|  |  | SI-127 |  |  | *Picrostigeus* sp. 1 |
|  |  | SI-128 |  |  | *Plectiscus* sp. 1 |
|  |  | SI-129 |  |  | *Plectiscus* sp. 2 |
|  |  | SI-130 |  |  | *Stenomacrus* sp. 2 |
|  |  | SI-131 |  |  | *Stenomacrus micropennis* |
|  |  | SI-132 |  |  | *Stenomacrus* sp. 1 |
|  |  | SI-133 |  | Braconidae | *Cotesia hallii* |
|  |  | SI-134 |  |  | *Dacnusa groenlandica* |
|  |  | SI-135 |  |  | *Praon brevistigma* |
|  |  | SI-136 |  |  | *Protapanteles fulvipes* |
|  |  | SI-137 |  | Apidae | *Bombus hyperboreus* |
|  |  | SI-138 |  |  | *Bombus polaris* |
|  |  | SI-139 |  | Encyrtidae | *Pseudencyrtus* sp. 1 |
|  |  | SI-140 |  | Eulophidae | *Aprostocetus meltoftei* |
|  |  | SI-141 |  | Pteromalidae | *Pachyneuron groenlandicum* |
|  |  | SI-142 | Lepidoptera | Geometridae | *Entephria punctipes* |
|  |  | SI-143 |  |  | *Entephria* sp. |
|  |  | SI-144 |  | Erebidae | *Gynaephora groenlandica* |
|  |  | SI-145 |  | Lycaenidae | *Plebeius glandon* |
|  |  | SI-146 |  | Noctuidae | *Apamea zeta* |
|  |  | SI-147 |  |  | *Euxoa adumbrata drewseni* |
|  |  | SI-148 |  |  | *Polia richardsoni* |
|  |  | SI-149 |  |  | *Sympistis zetterstedtii* |
|  |  | SI-150 |  |  | *Syngrapha parilis* |
|  |  | SI-151 |  | Nymphalidae | *Boloria chariclea* |
|  |  | SI-152 |  |  | *Boloria* spp. |
|  |  | SI-153 |  | Pieridae | *Colias hecla* |
|  |  | SI-154 |  | Pyralidae | *Pyla fusca* |
|  |  | SI-155 |  | Tortricidae | *Olethreutes mengelana* |

**References for Text S2**

Dormann, C.F., Fründ, J., Blüthgen, N. & Gruber, B. (2009) Indices, graphs and null models: Analyzing bipartite ecological networks. *The Open Ecology Journal,* 2**,** 7-24.

Elberling, H. & Olesen, J.M. (1999) The structure of a high latitude plant-flowe visitor system: the dominance of flies. *Ecography,* 22**,** 314-323.

Pocock, M.J.O., Evans, D.M. & Memmott, J. (2012) The robustness and restoration of a network of ecological networks. *Science,* 335**,** 973-977.

R, C.T. (2012) R: a language and environment for statistical computing. Vienna, Austria.

Rasmussen, C., Dupont, Y.L., Mosbacher, J.B., Trøjelsgaard, K. & Olesen, J.M. (2013) Strong Impact of Temporal Resolution on the Structure of an Ecological Network. *PLoS ONE,* 8**,** e81694.

Roslin, T., Wirta, H., Hopkins, T., Hardwick, B. & Várkonyi, G. (2013) Indirect interactions in the High Arctic. *PLoS ONE,* 8**,** e67367.
